# Gradient-based parameter optimization method to determine membrane ionic current composition in human induced pluripotent stem cell-derived cardiomyocytes

**DOI:** 10.1038/s41598-022-23398-0

**Published:** 2022-11-09

**Authors:** Hirohiko Kohjitani, Shigeya Koda, Yukiko Himeno, Takeru Makiyama, Yuta Yamamoto, Daisuke Yoshinaga, Yimin Wuriyanghai, Asami Kashiwa, Futoshi Toyoda, Yixin Zhang, Akira Amano, Akinori Noma, Takeshi Kimura

**Affiliations:** 1 Department of Cardiovascular Medicine, Kyoto University Graduate School of Medicine, Kyoto, Japan; 2 Graduate School of Life Sciences, Ritsumeikan University, Kusatsu, Japan; 3 Department Pediatrics, Kyoto University Graduate School of Medicine, Kyoto, Japan; 4 Department of Physiology, Shiga University of Medical Science, Otsu, Japan

**Keywords:** Computational biophysics, Cardiovascular biology

## Abstract

Premature cardiac myocytes derived from human induced pluripotent stem cells (hiPSC-CMs) show heterogeneous action potentials (APs), probably due to different expression patterns of membrane ionic currents. We developed a method for determining expression patterns of functional channels in terms of whole-cell ionic conductance (*G*_*x*_) using individual spontaneous AP configurations. It has been suggested that apparently identical AP configurations can be obtained using different sets of ionic currents in mathematical models of cardiac membrane excitation. If so, the inverse problem of *G*_*x*_ estimation might not be solved. We computationally tested the feasibility of the gradient-based optimization method. For a realistic examination, conventional 'cell-specific models' were prepared by superimposing the model output of AP on each experimental AP recorded by conventional manual adjustment of *G*_*x*_s of the baseline model. *G*_*x*_s of 4–6 major ionic currents of the 'cell-specific models' were randomized within a range of ± 5–15% and used as an initial parameter set for the gradient-based automatic *G*_*x*_s recovery by decreasing the mean square error (MSE) between the target and model output. Plotting all data points of the MSE–*G*_*x*_ relationship during optimization revealed progressive convergence of the randomized population of *G*_*x*_s to the original value of the cell-specific model with decreasing MSE. The absence of any other local minimum in the global search space was confirmed by mapping the MSE by randomizing *G*_*x*_s over a range of 0.1–10 times the control. No additional local minimum MSE was obvious in the whole parameter space, in addition to the global minimum of MSE at the default model parameter.

## Introduction

Over the past half-century, the biophysical characteristics of ion-transporting molecules (channels and ion exchangers) have been extensively analyzed. Biophysical models of each functional component have largely been detailed^1–4^ , including human induced pluripotent stem cells (hiPSC-CMs)^5–7^. In addition, various composite cell models, including membrane excitation, cell contraction, and intracellular ionic composition homeostasis, have been developed by integrating mathematical models at the molecular level into cardiac cell models^8–11^. These models have been useful for visualizing individual currents underlying the action potential (AP) configuration under various experimental conditions in mature cardiac myocytes. However, the utility of these mathematical cell models is limited because of the lack of extensive validation of the model output accuracy. This is a drawback of the subjective manual fitting method used in almost all published mathematical cardiac cell models. A new challenge of mechanistic models of cardiac membrane excitation might be an examination in a very different paradigm to assess if the many, but continuous, variety of cardiac AP configurations, such as those recorded in hiPSC-CMs, can be reconstructed by applying the automatic parameter optimization method to the hiPSC-CM version of human cardiac cell models. We do not intend to propose a new hiPSC-CM model.

The automatic parameter optimization technique objectively determines parameters in a wide range of biological models, including cardiac electrophysiology^12–15^, systems pharmacology^16–20^, and other models. Because of this utility, many improvements in information technology have been realized^21,22^. However, in electrophysiology, different combinations of model parameters may produce very similar APs^13,23–25^. The determination of current density at high fidelity and accuracy likely requires additional improvements to the optimization method in the cardiac cell model because of the complex interactions among ionic currents underlying membrane excitation^23,26^.

The final goal of our study is to develop an objective and accurate method for determining the current profile (i.e., the expression level of functional ionic currents) underlying individual AP configurations. As a case study, we chose a large variety of AP configurations in hiPSC-CMs, which are difficult to classify into the conventional nodal, atrial, or ventricular types. The molecular bases of the ion channels expressed in hiPSC-CMs correspond to those in adult cardiac myocytes in the GSE154580 Gene Expression Omnibus Accession viewer. Electrophysiological findings suggest that the gating of ionic currents is quite similar to that observed in mature myocytes^27^. Thus, we modified the ion channel gating kinetics of the human ventricular cell (hVC) model^11^ according to the prior experimental measurements^27^ for a hiPSC-CM type baseline model of the parameter optimization (PO) method. For simplicity, we assumed that the opening/closing kinetics of ion channels expressed by the same human genome remains the same among hiPSC-CMs. We also assumed that the heterogeneity of the electrical activities of hiPSC-CMs might be determined by the variable expression levels of ion channels in the cell membrane. We computationally examined the feasibility of one of the basic gradient-based optimization methods, the pattern search (PS) algorithm^21,22,28^, in a model of cardiac AP generation. We prepared a given AP configuration using each 'cell-specific model' prepared by the conventional manual fitting of the hVC model to the respective experimental recordings. To assess the accuracy of the PS method for parameter optimization, the AP waveform generated by the cell-specific model was used as a target of the optimization. The initial set of parameters for the optimization was then prepared by uniform randomization centered around the default values of the model. The PS algorithm should return the original parameter values by decreasing the mean squared error (MSE) function between the modified model output and target AP waveforms. The accuracy of the optimization was determined by recovering the original values of each ionic current amplitude as the MSE progressively decreased toward zero.

## Materials and Methods

### Baseline model of hiPSC-CM membrane excitation

The baseline model of hiPSC-CMs was essentially the same as the hVC model, which has been detailed^10,11^ and which shares many comparable characteristics with other published human models^8,9^. The hVC model consists of a cell membrane with a number of ionic channel species and a few ion transporters, the sarcoplasmic reticulum equipped with the Ca^2+^ pump (SERCA), and the refined Ca^2+^ releasing units coupled with the L-type Ca^2+^ channels on the cell membrane at the nanoscale dyadic space^4,29^, contractile fibers, and three cytosolic Ca^2+^ diffusion spaces containing several Ca^2+^-binding proteins (Fig. S1). All model equations and abbreviations are described in the Supplemental Materials.

The source code of the hiPSC-CM model was written in VB.Net and is available from the archive site (https://doi.org/10.1101/2022.05.16.492203).

The kinetics of the ionic currents in the baseline model were readjusted according to new experimental measurements if available in hiPSC-CMs^27^ (Fig. S2). In the present study, the net membrane current (*I*_*tot_cell*_) was calculated as the sum of nine ion channel currents and two ion transporters (*I*_*NaK*_ and *I*_*NCX*_) (Eq. 1).

1 $$ \begin{array}{*{20}c} {{\text{I}}_{{{\text{tot}}\_{\text{cell}}}} = {\text{I}}_{{{\text{Na}}}} + {\text{I}}_{{{\text{CaL}}}} + {\text{I}}_{{{\text{ha}}}} + {\text{I}}_{{{\text{K}}1}} + {\text{I}}_{{{\text{Kr}}}} + {\text{I}}_{{{\text{Ks}}}} + {\text{I}}_{{{\text{Kur}}}} + {\text{I}}_{{{\text{Kto}}}} + {\text{I}}_{{{\text{bNSC}}}} + {\text{I}}_{{{\text{NaK}}}} + {\text{I}}_{{{\text{NCX}}}} } \\ \end{array} $$

The membrane excitation of the model is generated by charging and discharging the membrane capacitance (*C*_*m*_) using the net ionic current (*I*_*tot_cell*_) across the cell membrane (Eq. 1). The driving force for the ionic current is given by the potential difference between *V*_*m*_ and the equilibrium potential (*E*_*x*_) (Eq. 3). The net conductance of the channel is changed by the dynamic changes in the open probability (*pO*) of the channel, which is mostly *V*_*m*_-dependent through the *V*_*m*_-dependent rate constants ($\alpha , \beta$) of the opening and closing conformational changes of the channel (Eq. 4 and 5).

2 $$ \begin{array}{*{20}c} {\frac{{dV_{m} }}{dt} = - \frac{{I_{tot\_cell} }}{{C_{m} }} = - \frac{{\sum I_{x} }}{{C_{m} }} } \\ \end{array} $$

3 $$ \begin{array}{*{20}c} {I_{x} = \overline{G}_{x} \cdot pO \cdot \left( {V_{m} - E_{x} } \right) } \\ \end{array} $$

4 $$ \begin{array}{*{20}c} {\frac{dpO}{{dt}} = \alpha \cdot \left( {1 - pO} \right) - \beta \cdot pO} \\ \end{array} $$

5 $$ \begin{array}{*{20}c} {\left[ {\alpha \beta } \right]^{T} = f\left( {V_{m} } \right) } \\ \end{array} $$

The exchange of three Na^+^ / two K^+^ by the Na/K pump, and three Na^+^ / one Ca^2+^ exchange by sodium-calcium exchanger (NCX) also generates sizeable fractions of membrane ionic current (*I*_*NaK*_, and *I*_*NCX*_, respectively). For simplicity, we excluded background currents of much smaller amplitude, such as *I*_*KACh*_*, I*_*KATP*_*, I*_*LCCa*_, and *I*_*Cab*_, from the parameter optimization and adjusted only the non-selective background cation current (*I*_*bNSC*_) of significant amplitude^30–32^. In the present study, *I*_bNSC_ is re-defined as a time-independent net current, which remains after blocking all time-dependent currents.

### Computational parameter optimization

The whole-cell conductance *G*_*x*_ of a given current system (*x*) is modified by multiplying the limiting conductance ${\overline{G} }_{x}$ (Eq. 3) of the baseline model by a scaling factor *sf*_*x*_ (Eq. 6) and is used for the parameter optimization.

6 $$ \begin{array}{*{20}c} {G_{x} = \overline{G}_{x} \cdot sf_{x} } \\ \end{array} $$

The MSE function (Eq. 7) was used in the parameter optimization, where *V*_*m,a*_ represents the adaptive *V*_*m*_ (the model output) generated by adjusting the *sf*_*x*_s of the baseline model. Target *V*_*m,t*_ represents the AP of the intact baseline model.

7 $$ \begin{array}{*{20}c} {MSE = \frac{{\sum \left( {V_{m,a} - V_{m,t} } \right)^{2} }}{N}} \\ \end{array} $$

The MSE was stabilized by obtaining a quasi-stable rhythm of spontaneous APs through continuous numerical integration of the model. Typically, 30–100 spontaneous cycles were calculated for a new set of *sf*_*x*_s. The MSE was calculated within the time window. The width of the time window was adjusted according to the AP phase of interest. where N is the number of digitized *V*_*m*_ points with a time interval of 0.1 ms.

In typical parameter optimization, *V*_*m,a*_ is generated by modifying the baseline model for comparison with the experimental record (*V*_*m,t*_ = *V*_*m,rec*_). However, to evaluate the identifiability of the parameter optimization, a simple approach was adopted in the present study. We used the manually adjusted 'cell-specific' model for the target (*V*_*m,t*_), which was nearly identical to *V*_*m,rec*_. More importantly, the 'cell-specific' *V*_*m*_ is completely free from extra fluctuations (noise), which were observed in almost all AP recordings in hiPSC-CMs. In the optimization process, the initial value of each optimization parameter was prepared by randomizing the *sf*_*x*_s of the cell-specific model by ± 5–15% at the beginning of each run of PS (*V*_*m,orp*_) in Eq. 8, and several hundred PS runs were repeated. Thus, the error function is

8 $$ \begin{array}{*{20}c} {MSE = \frac{{\sum \left( {V_{m,orp} - V_{m,t} } \right)^{2} }}{N} } \\ \end{array} $$

This optimization method was termed the 'orp test' in the present study.

The advantage of using a manually adjusted cell model for the optimization target is that the accuracy of parameter optimization is proved by recovering all *sf*_x_ = 1 (Eq. 6) independent from the randomized initial parameter set. The same approach was used in a previous study^23^ to evaluate the accuracy of parameter optimization by applying the genetic algorithm to the TNNP model of the human ventricular cell^33^.

Optimization using the randomized initial model parameters was repeated for more than 200 runs. Thus, the orp test might be classified in a 'multi-run optimization'. The distribution of the *sf*_x_ data points obtained during all test runs was plotted in a single *sf*_x_-MSE coordinate to examine the convergence of individual *sf*_x_s with the progress of the orp test.

### Pattern Search method for parameter optimization

For a system showing a relatively simple gradient of MSE along the parameter axis, gradient-based optimization methods are generally more efficient than stochastic methods for this type of objective function. We used the Pattern Search (PS) algorithm, a basic gradient-based optimization method. The computer program code for the PS^34^ is simple (see Supplemental Materials) and does not require derivatives of the objective function. We implemented the code in a homemade program for data analysis (in VB) to improve the method for better resolution and to save computation time.

The primary PS method uses the base and new points^28^. Briefly, *sf*_x_ is coded with symbols *BP*_x_ and *NP*_x_ in the computer program, representing a base point (*BP*_x_) and a new search point (*NP*_x_), respectively. The MSE is calculated for each movement of *NP*_x_ by adding or subtracting a given step size (*stp*) to the *BP*_x_, and the search direction is determined by the smaller MSE. Then, the entire mathematical model is numerically integrated (Eq. 2–5) using *NP*_x_ to reconstruct the time course of AP (*V*_m,a_). This adjustment is performed sequentially for each of the four to six selected currents in a single optimization cycle. The cycle is repeated until no improvement in the MSE was achieved by a new set of *NP*_x_s. The *BP*_x_ set is then renewed by the new set of *NP*_x_ for the subsequent series of optimizations. Simultaneously, *stp* is reduced by a given reduction factor (*redFct* of 1/4). The individual PS run is continued until the new *stp* became smaller than the critical *stp* (*crtstp*), which was set to 2–10 × 10^–5^ in the present study.

### Selection of ionic currents for the optimization

When we obtain a new experimental record of AP, we do not start the analysis with an automatic optimization of *G*_*x*_. Rather, we first adjust the baseline model by conducting conventional manual fitting. The nine ionic currents in Eq. 1 in the baseline model are adjusted incrementally to superimpose the simulated AP on the experimental one. During this step, it is important to pay attention to the influence of each *sf*_*x*_ adjustment on the simulated AP configuration on the computer display. Thereby, one may find several key current components that should be used for the automatic parameter optimization. Usually, currents showing a relatively large magnitude of *G*_*x*_ were selected for automatic optimization according to Eq. 1, while those that scarcely modified the simulated AP were left as default values in the baseline model.

### Principal component analysis of cell-specific models

When the orp test was performed with *p* elements, it was possible to record the final point BP, where the MSE was improved in the *p*-dimensional space. Suppose that we represent the matrix when *n* data points are acquired as an *n* × *p* matrix *X*. In that case, we obtain a vector space based on the unit vector that maximizes the variance (first principal component: PC1) and the *p*-dimensional unit vector orthogonal to it (loadings vector ${{\varvec{w}}}_{\left(k\right)}=\left({w}_{1},{w}_{2}\cdots ,{w}_{p}\right))$. It is possible to convert each row ${{\varvec{x}}}_{\left(i\right)}$ of the data matrix *X* into a vector of principal component scores ${{\varvec{t}}}_{\left(i\right)}$. The transformation is defined as

9 $$ \begin{array}{*{20}c} {{\varvec{t}}_{k\left( i \right)} = {\varvec{x}}_{\left( i \right)} \cdot {\varvec{w}}_{\left( k \right)} for i = 1,2, \cdots ,n k = 1,2, \cdots ,p } \\ \end{array} $$

To maximize the variance, the first weight vector w_(1)_ corresponding to the first principal component must satisfy:

10 $$ \begin{array}{*{20}c} {{\varvec{w}}_{\left( 1 \right)} = \arg \mathop {\max }\limits_{{\varvec{w}}} \left\{ {\frac{{{\varvec{w}}^{T} {\varvec{X}}^{T} {\varvec{Xw}}}}{{{\varvec{w}}^{T} {\varvec{w}}}}} \right\}} \\ \end{array} $$

The kth component can be determined by subtracting the first (k-1)-th principal components from X:

11 $$ \begin{array}{*{20}c} {\widehat{{X_{k} }} = X - \mathop \sum \limits_{s = 1}^{k - 1} {\varvec{Xw}}_{\left( s \right)} {\varvec{w}}_{\left( s \right)}^{T} } \\ \end{array} $$

The weight vector is then given as a vector such that the variance of the principal component scores is maximized for the new data matrix:

12 $$ \begin{array}{*{20}c} {{\varvec{w}}_{\left( k \right)} = \arg \mathop {\max }\limits_{{\varvec{w}}} \left\{ {\frac{{{\varvec{w}}^{T} \hat{\user2{X}}_{{\varvec{k}}}^{T} \hat{\user2{X}}_{k} {\varvec{w}}}}{{{\varvec{w}}^{T} {\varvec{w}}}}} \right\}} \\ \end{array} $$

### Membrane excitation and its cooperativity with intracellular ionic dynamics

When any of the *G*_*x*_s is modified, the intracellular ion concentrations ([ion]_i_) change, although the variation is largely compensated for with time in intact cells by modifying the activities of both the three Na^+^ / two K^+^ pump (NaK) and three Na^+^ / one Ca^2+^ exchange (NCX). In the present study, we imitated the long-term physiological homeostasis of [ion]_i_ by introducing empirical Eq. 13 and 14. These equations induced 'negative feedback' to the capacity (max*I*_*NaK*_ and max*I*_*NCX*_) of these ion transporters. Each correcting factor (*crf*_*x*_) was continuously scaled to modify the limiting activity of the transporters to maintain the [Na^+^]_i_ or the total amount of Ca within the cell (Ca_tot_) equal to their pre-set level (*std*_*Nai*_, *std*_*Catot*_) with an appropriate delay (coefficients 0.3 and 0.008 in Eq. 13 and 14, respectively).

For the control of [Na^+^]_i_:

13 $$ \begin{array}{*{20}c} {\Delta crf_{NaK} = - \left( {stdNa_{i} - Na_{i} } \right) \times 0.3, \,stdNa_{i} = 6.1mM, } \\ {I_{NaK} = \left( {crf_{NaK} \cdot maxI_{NaK} } \right) \cdot \nu cyc_{NaK} } \\ \end{array} $$

For the control of Ca_tot_:

14 $$ \begin{array}{*{20}c} {\Delta crf_{NCX} = - \left( {stdCa_{tot} - Ca_{tot} } \right) \times 0.008, \,stdCa_{tot} = 79amol, } \\ {I_{NCX} = \left( {crf_{NCX} \cdot maxI_{NCX} } \right) \cdot \left( {k_{1} \cdot E_{1Na} \cdot E_{1NCX} - k_{2} \cdot E_{2Na} \cdot E_{2NaCa} } \right) } \\ \end{array} $$

Ca_tot_ is given by [Ca]_i_ included in the cytosolic three Ca-spaces *jnc*, *iz*, and *blk,* and in the sarcoplasmic reticulum *SR*_*up*_ and *SR*_*rl*_ in the free or bound forms, respectively.

15 $$ \begin{array}{*{20}c} { Ca_{tot} = \left[ {Ca_{tot} } \right]_{jnc} \cdot vol_{jnc} + \left[ {Ca_{tot} } \right]_{iz} \cdot vol_{iz} + \left[ {Ca_{tot} } \right]_{blk} \cdot vol_{blk} + } \\ \end{array} \left[ {Ca_{tot} } \right]_{SRup} \cdot vol_{SRup} + \left[ {Ca_{tot} } \right]_{SRrl} \cdot vol_{SRrl} $$

*where* vol *is the volume of the cellular Ca compartment (see more details*^11^*).*

### Preparation of dissociated hiPSC-CMs and recording of spontaneous APs

The 201B7 and 253G1 hiPSC lines generated from healthy individuals were used in this study^35,36^. The differentiation of hiPSCs into cardiomyocytes was promoted using an embryoid body differentiation system^37^. The hiPSCs were incubated at 37 °C in 5% CO_2_, 5% O_2_, and 90% N_2_ for the first 12 days to promote differentiation. hiPSCs aggregated to form embryoid bodies and were cultured in suspension for 20 days. On day 20 of culture, embryoid bodies were treated with collagenase B (Roche, Basel, Switzerland) and trypsin–EDTA (Nacalai Tesque, Kyoto, Japan) and dispersed into single cells or small clusters, which were plated onto 0.1% gelatin-coated dishes. hiPSC-CMs were maintained in a conditioned medium. The experimental study using hiPSC-CMs was approved by the Kyoto University ethics review board (G259) and conformed to the principles of the Declaration of Helsinki.

### Electrophysiological recordings of hiPSC-CM APs

For single-cell patch-clamp recordings, gelatin-coated glass coverslips were placed into each well of a 6-well plate. Two milliliters of DMEM/F12 containing 2% fetal bovine serum and 80,000–120,000 CMs were added to each well. Spontaneous APs were recorded from beating single CMs using the perforated patch-clamp technique with amphotericin B (Sigma-Aldrich, St. Louis, MO, USA) at 36 ± 1 ºC. Data were acquired at 20 kHz using a Multiclamp 700 B amplifier (Molecular Devices, Sunnyvale, CA, USA), Digidata 1440 digitizer hardware (Molecular Devices), and pClamp 10.4 software (Molecular Devices). The glass pipettes had a resistance of 3–6 MΩ after being filled with the intracellular solution. The external solution used for AP recordings was composed of (in mM): NaCl 150, KCl 5.4, CaCl_2_ 1.8, MgCl_2_-6H_2_O 1, glucose 15, HEPES 15, and Na-pyruvate 1. The pH was adjusted to 7.4 by titrating with NaOH. Intracellular solution contained (in mM): KCl 150, NaCl 5, CaCl_2_ 2, EGTA 5, MgATP 5, and HEPES 10 (pH adjusted to 7.2 with KOH), as well as amphotericin B 300 µg/ml.

## Results

### Mapping the magnitude of MSE over the nine global parameter space

**Figure 1:**
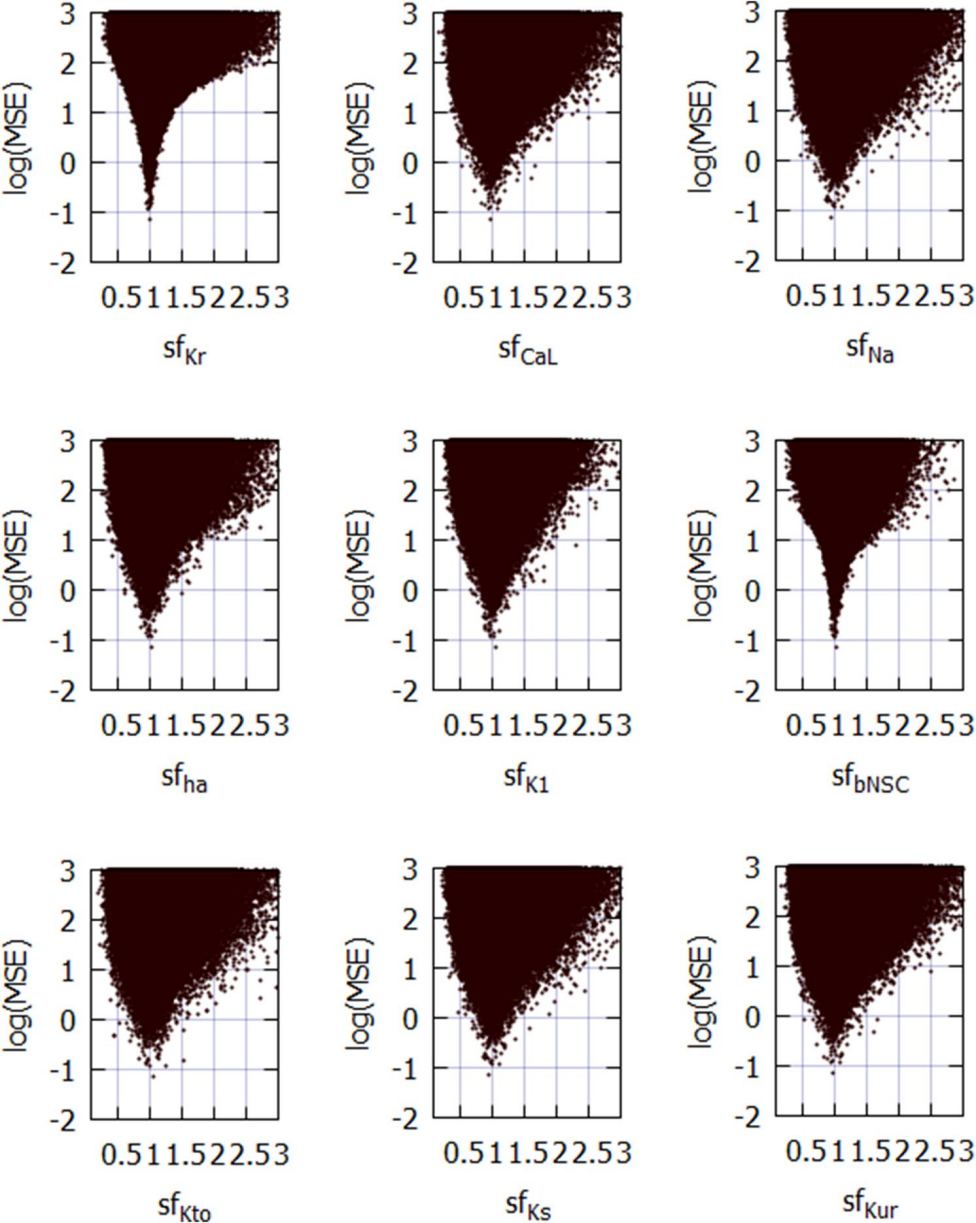
Distribution of MSE calculated between the target and simulated APs modified by randomizing the *sf*_*x*_ of nine ionic currents in coordinates of MSE-*sf*_*x*_. All MSE data points were plotted on the logarithmic ordinate against the linear *sf*_*x*_. A total of 5,141,382 points were calculated in cell model No. 86 over the range of 1/10 to approximately 10 times the default *sf*_*x*_. Since the configuration of *V*_*m*_ records were largely unrealistic at *sf*_*x*_ > 3, MSE points were omitted over *sf*_*x*_ > 3.0. To demonstrate the sharp decrease in MSE, the data points were densely populated near the default *sf*_*x*_.

Parameter identifiability is a central issue in the parameter optimization of biological models^14,20^. To confirm the identifiability of a unique set of *sf*_*x*_s using the parameter optimization method, mapping of the MSE distribution over an enlarged parameter space defined by the *sf*_*x*_ of the nine ionic currents of the baseline model is required. The randomization of *sf*_*x*_ ranged from 1/10 to approximately 10 times the default values. The calculation was performed for approximately 5,000,000 sets, as shown in Fig. 1; magnitudes of $log\left(MSE\right)$ were plotted against each *sf*_*x*_ on the abscissa.

The data points of MSE at a given *sf*_*x*_ include all variable combinations of the other eight *sf*_*x*_s. The algorithm of the PS method searches for a parameter set, which gives the minimum MSE at a given *stp* through the process of optimization. Drawing a clear envelope curve by connecting the minimum MSEs at each *sf*_*x*_ was difficult because of the insufficient number of data points in these graphs (Fig. 1). Nonetheless, an approximate envelope of the minimum MSEs may indicate a single global minimum of MSE located at the control *sf*_*x*_ equals one, as typically exemplified by *sf*_*Kr*_- and *sf*_*bNSC*_-MSE relations. On both sides of the minimum, steep slopes of MSE/*sf*_*x*_ were evident in all graphs. Outside this limited *sf*_*x*_ -MSE area, the global envelope showed a gentle and monotonic upward slope toward the limit on the right side. No local minimum was observed in all of the *sf*_*x*_ -MSE diagrams, except for the central sharp depression. Essentially the same finding was obtained in another hiPSC-CM model (Cell 38), which showed less negative MDP (see Fig. S4 in Supplemental Materials). It was concluded that the theoretical model of cardiac membrane excitation (hVC model) has only a single central sharp depression corresponding to the control model parameter.

### Necessity for parameter optimization as indicated by hiPSC-CM APs

**Figure 2:**
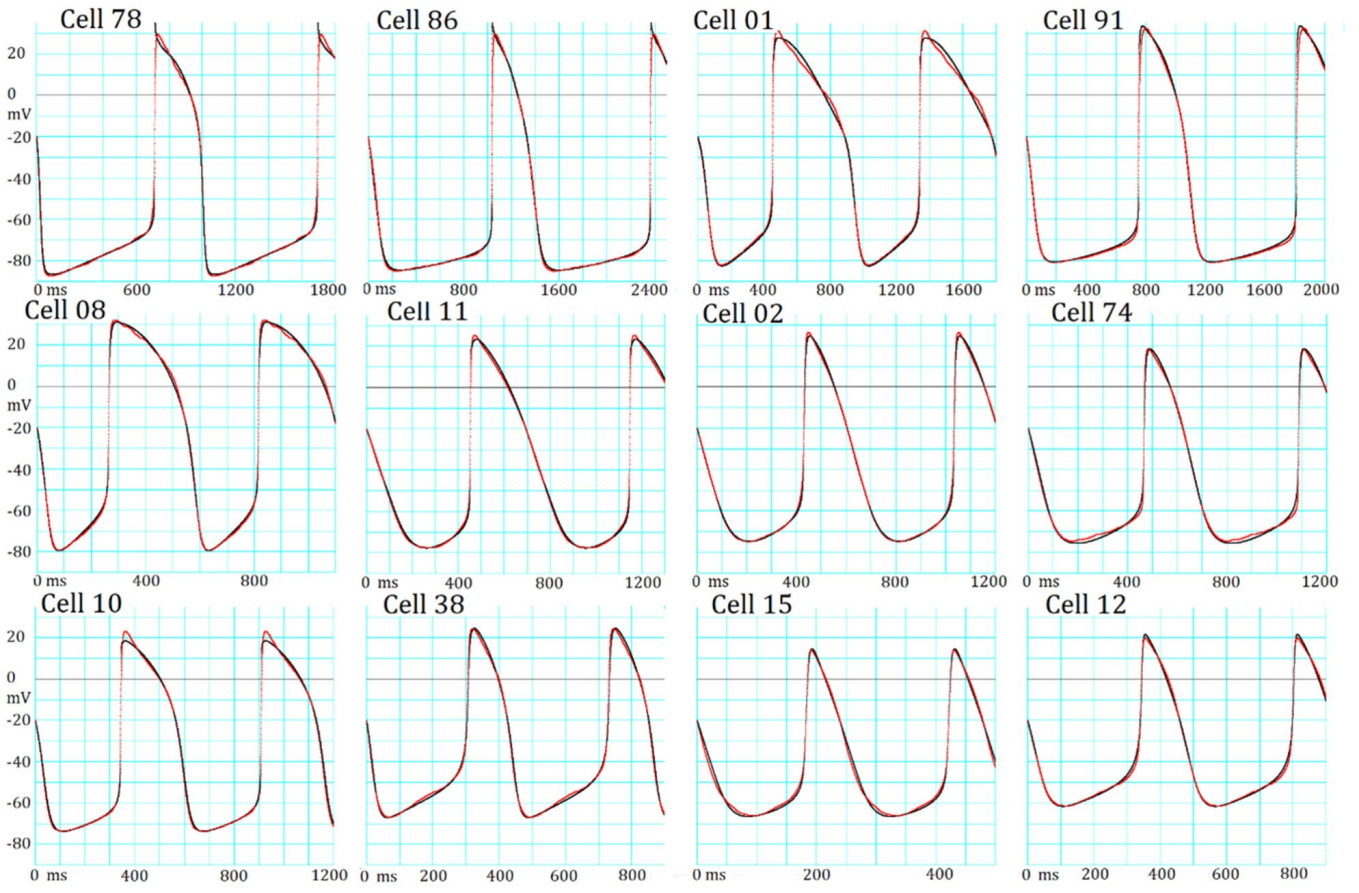
Manual fitting of variable AP configurations in 12 different hiPSC-CMs. Each panel shows the experimental record (red) superimposed by the model output (black) of the baseline model adjusted by the conventional manual fitting. The experimental cell number is presented at the top of each pair of AP records, The extra fluctuations are obvious during the AP plateau in Cells 78, 08, and 01, and during SDD in Cells 15 and 74. The length of abscissa is markedly different to illustrate the interval between two successive peaks of AP.

Figure 2 illustrates the records of spontaneous APs (red traces) obtained from 12 experiments in the maximum diastolic potential (MDP) sequence (see Supplemental Materials for details). All experimental records were superimposed with simulated AP traces (black traces) obtained using conventional manual fitting. In most cases, an MSE of 1–6 mV^2^ remained (Eq. 7) at the end of the manual fitting. This extra component of MSE might be largely attributed to slow fluctuations of *V*_*m*_ of unknown origin in experimental recordings, because the non-specific random fluctuations were quite different from the exponential gating kinetics of ion channels calculated in mathematical models. This extra noise seriously interfered with the assessment of the accuracy of the parameter optimization of *G*_*x*_ in the present study. Thus, APs produced by the manual adjustment (cell-specific model) was used as the target AP that was completely free from the extra noise when examining the feasibility of the parameter optimization algorithm.

Comparison of the AP configurations between these hiPSC-CMs clearly indicated that the classification of these APs into atrial, ventricular, and nodal types was impractical, as has been described^7^. On the other hand, if provided with the individual models fit by objective parameter optimizing tools using the baseline model (black trace), the results should be fairly straightforward for estimating the functional expression level of ion channels and to clarify the role of each current system or the ionic mechanisms in generating the spontaneous AP configuration in a quantitative manner. Thus, the objective parameter optimization of the mathematical model is a vital requirement in cardiac electrophysiology.

Table 1 lists the AP metrics, including cycle length (CL), peak potential of the plateau (OS), MDP, and AP duration measured at -20 mV in addition to the MSE between individual experimental records and the model output fitted by manual fitting. CL, MDP, and AP varied markedly among different AP recordings of cells (Fig. 2). Cells were arranged according to the MDP sequence.

**Table 1:** AP metrics and MSE calculated after manual fitting of the various AP configurations in 12 different hiPSC-CMs shown in Fig. 2.

Cell	CL (ms)	OS (mV)	MDP (ms)	APD at − 20 mV (ms)	MSE (mV^2^)
Cell 78	983.8	29.6	− 87.4	271.7	5.8443
Cell 86	1326.0	29.7	− 85.0	289.2	4.0554
Cell 01	887.4	31.2	− 82.2	435.0	3.9330
Cell 91	1058.0	33.0	− 80.4	308.6	7.2156
Cell 08	551.4	32.0	− 79.5	287.6	1.4043
Cell 11	695.0	25.3	− 77.6	243.5	2.6683
Cell 02	603.9	26.4	− 74.9	173.4	1.0412
Cell 74	622.8	18.5	− 74.8	157.0	2.2589
Cell 10	564.3	23.0	− 73.7	220.9	3.2194
Cell 38	425.4	24.2	− 66.8	123.4	3.6626
Cell 15	239.5	13.8	− 66.1	57.1	2.8607
Cell 12	458.6	19.7	− 61.5	119.0	1.3514

### Feasibility of PS algorithm for parameter optimization of membrane excitation models

Automatic parameter optimization has been applied to the model of cardiac membrane excitation in a limited number of studies (for reviews, see^23,26,38,39^) using various optimization methods, such as genetic algorithms. To the best of our knowledge, the principal PS algorithm has not been successfully applied to detailed mathematical models of cardiac membrane excitation composed of both ionic channel and ion transporter models, except for one pioneering study^12^, which applied a more general gradient-based optimization method to the simple ventricular cell model (Beeler and Reuter [BR] model)^40^.

**Figure 3:**
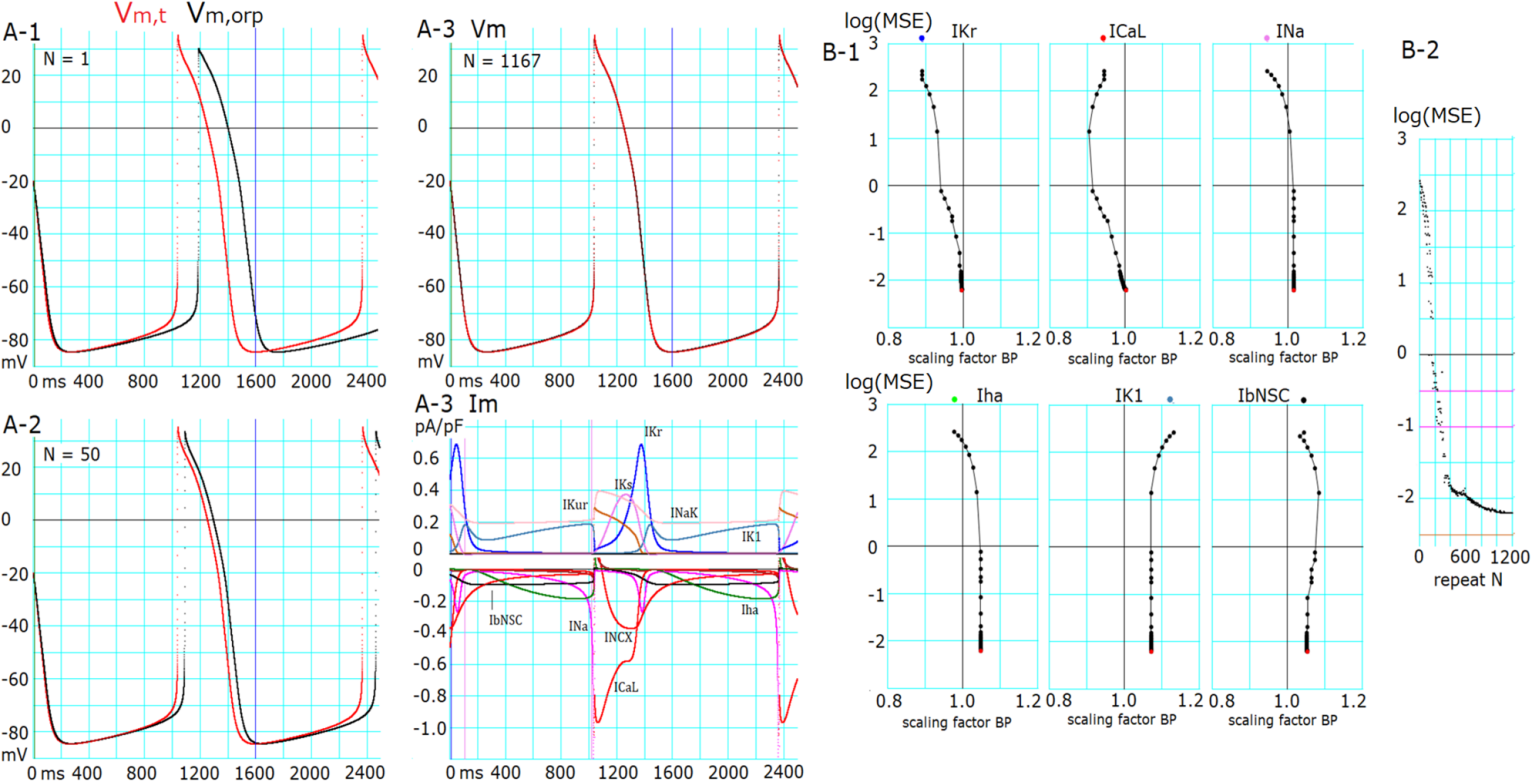
Successful optimization in Cell 86. (**A-1**) Target AP (*V*_*m,t*_, red) and AP generated by randomized initial *sf*_*x*_s (*V*_*m,orp*_, black). (**A-2**) *V*_*m,t*_ (red) and *V*_*m,orp*_ (black) generated after 50 cycles of adjusting BP. (**A-3**) *V*_*m*_: *V*_*m,t*_ (red) and *V*_*m,orp*_ (black) generated by the final *sf*_*x*_s. *I*_*m*_: corresponding time courses of each current for the finalized AP shown in (**A-3**) *V*_*m*_. (**B-1**) Changes in *sf*_*x*_s vs. log(MSE) during a successful optimization process of PS. (**B-2**) log(MSE) of all BP points during the search process in PS. The initial values of *sf*_*x*_s are plotted by corresponding colors at the top of each *sf*_*x*_-log(MSE) graph.

Figure 3 shows a typical successful run of the new PS method in hiPSC-CMs. An approximate MDP of −85 mV was evident. The PS parameter optimization was started after randomizing the *sf*_*x*_s of the six major currents (*I*_*Kr*_, *I*_*CaL*_, *I*_*Na*_, *I*_*ha*_, *I*_*K1*_ and *I*_*bNSC,*_) in the manual fit model within a range of ± 15% around the default values (normalized magnitude of 1). Figure 3A 1-3-compare the simulated *V*_*m,orp*_ (black) with the target *V*_*m,t*_ (red) at the repeat numbers of N = 1, 50, and 1167, respectively (Eq. 8). The overshoot potential (OS), APD, and CL of spontaneous AP were markedly different during the first cycle of AP reconstruction (Fig. 3A-1). These deviations were largely decreased during the PS cycle (; Fig. 3A-1; *V*_*m*_ at N = 50) and became invisible in the final result (Fig. 3A-3; N = 1167). The final individual current flows of the nine current components (*I*_*m*_) are shown in the lower panel of Fig. 3A-3.

The time course of decreasing $log\left(MSE\right)$ evoked by the multi-run PS optimization was plotted for each *sf*_*x*_ every time the set of base points was reset (Fig. 3B-1).Figure 3B-2 shows all of the $log\left(MSE\right)$ obtained at every adjustment by stepping individual BP points. The movements of all *sf*_*x*_s were synchronized to decrease $log\left(MSE\right)$ from approximately 2.4–1 during the initial 180 cycles of decreasing $log\left(MSE\right)$. However, the search directions of BP were quite variable. It seems that the detailed automatic adjustment of *sf*_*x*_s below $log\left(MSE\right)<0$ was driven by adjusting *I*_*Kr*_, *I*_*CaL*_, and *I*_*bNSC*_ in this cell. The values of *sf*_*Kr*_, *sf*_*CaL*_, and *sf*_*Na*_ approached the correct value of 1, whereas those for *I*_*ha*_, *I*_*K1*_, and *I*_*bNSC*_ deviated from the unit by < 10% of the value. The explanation of the deviation of these three *sf*_*x*_s from the unit was examined and is presented in the next section.

### Successful determination of conductance parameters of membrane excitation models using the six-parameter optimization of randomized model parameters (orp) test

**Figure 4:**
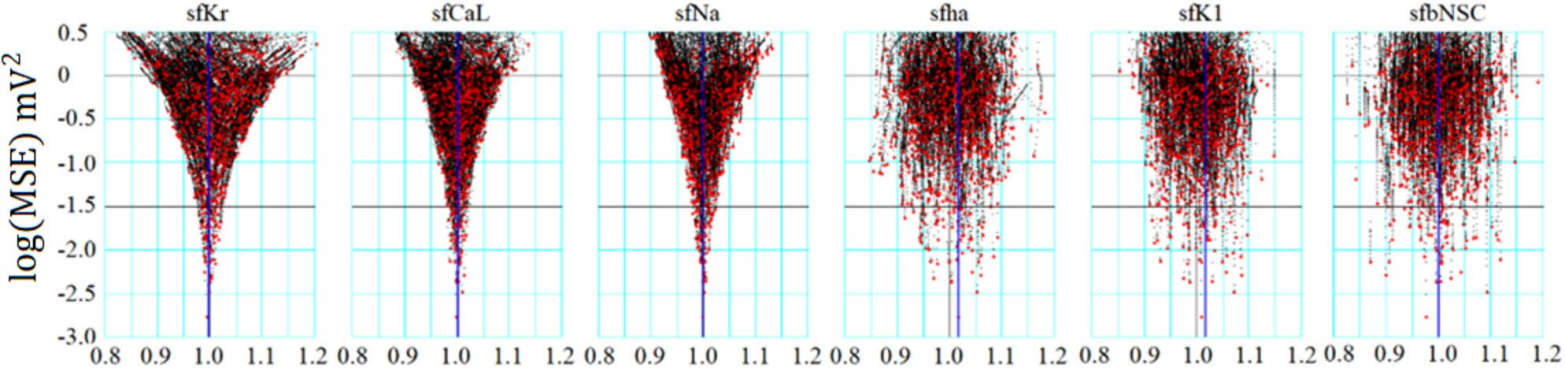
Convergence of *sf*_*x*_ in the orp test for Cell86. The ordinate is the *log(MSE)* and the abscissa is the normalized amplitude of *sf*_*x*_. *x* denotes *Kr*, *CaL*, *Na*, *ha*, *K1*, and *bNSC*. Black points were obtained in the progress of optimization, and red points are the final points in 829 runs of PS optimization.

In individual runs, the PS optimization was frequently interrupted at intermediate levels during the progress of optimization and the probability of reaching $log\left(MSE\right)$, for example, below – 2, rapidly decreased with increasing extent of randomization of the initial set of parameters. Moreover, the complementary relationships between several ionic currents in determining d*V*_*m*_/dt might have hampered parameter optimization. These facts indicate the need for statistical measures to evaluate the accuracy of the PS method. Figure 4 shows the results of the orp tests, in which the optimization shown in Fig. 3 was repeated several hundred times. All results were plotted in a common coordinate of $log\left(MSE\right)$ and individual *sf*_*x*_s. The population of *sf*_*x*_ correctly converged at a single peak point very close to 1 with increasing negativity of $log\left(MSE\right)$ for *sf*_*Kr*_, *sf*_*CaL*_, and *sf*_*Na*_. In contrast, *sf*_*ha,*_* sf*_*K1,*_ and *sf*_*bNSC*_ showed obvious variance. Nevertheless, they also showed a clear trend toward convergence to 1 on average. We could find clear convergence of less number (4) of *sf*_*x*_s used for PO method in cells, which showed relatively low MDPs as shown in the supplemental Materials (Fig. S5).

Table 2 summarizes the mean *sf*_*x*_ determined for the top 20 runs of PS parameter optimization in each of the 12 cells illustrated in Fig. 2. [Na^+^]_i_ and Ca_tot_ were well controlled to the reference levels (*std*_*Nai*_, and *std*_*Catot*_ in Eq. 16 and 17), with respective values of 6.1 mM and 79 amol, at the end of the parameter optimization to ensure constant [Na^+^]_i_ and Ca_tot_. The mean final $log\left(MSE\right)$ of –2.74 indicates that the MSE was reduced by five orders of magnitude from the initial level just after randomization by the orp test, as in the successful example shown in Fig. 3B. The mean of individual *sf*_*x*_s was very close to 1 with a minimum standard error (SE) of the mean, which was < 1% of the mean, even for *I*_*K1*_, *I*_*bNSC*_, and *I*_*ha*_, which showed weak convergence against $log\left(MSE\right)$. These results validate the accuracy of the parameter optimization using the multi-run PS method in all 12 cell-specific models, which include many varieties of spontaneous AP recorded in hiPSC-CMs.

**Table 2:** Measurements of *sf*_*x*_s (mean + SE, N = 20), [Na^+^]_i_ (mM), and Ca_tot_ (amol) in the 12 cells.

Cell No	log(MSE)	sfKr	sfK1	sfCaL	sfbNSC	sfha	sfNa	sfKur	[Na^+^]_i_(mM)	Ca_tot_(amol)
78	− 2.48321	1.00005	1.00157	1.00037	0.99460	1.00060	1.00134		6.10550	78.99979
91	− 2.42008	0.99952	1.00644	1.00063	1.00280	1.00470	1.00068		6.09977	79.00044
86	− 2.80257	1.00166	1.01394	1.00142	1.02670	1.00253	1.00702		6.09466	79.00008
01	− 2.79709	0.99871	1.00157	0.99756	0.99692	1.00054	0.99779		6.08973	78.99984
08	− 3.07432	0.00094	0.99982	1.00088	1.00041	0.99968	0.99985		6.12201	79.00056
11	− 2.67641	1.00186	1.00686	1.00129	0.99768	1.00253	1.01028		6.10385	78.99995
10	− 1.70278	1.00322	1.01081	1.00424	1.00396		0.99883		6.10968	79.00018
02	− 2.35441	1.00161	1.02038	1.00341	0.99815	1.01324	1.00954		6.10184	79.00004
74	− 2.43399	1.00126	1.01838	1.00308	0.99898	1.00004	1.00435		6.10118	79.99979
38	− 3.01883	1.00075		1.00106	1.00061		0.98866	1.00151	6.10530	78.99969
15	− 3.85992	1.00003		0.99894	0.99996		1.00015	0.98653	6.09902	79.00022
12	− 3.33037	0.99978		1.00030	0.99990	0.97587		1.00188	6.10012	79.00007
Ave	− 2.74617	0.99992	1.00886	1.00110	1.001723	0.99997	1.001681	0.99664	6.10272	79.08339
SE	0.07065	0.00093	0.010000	0.00164	0.00430	0.00729	0.00544	0.00690	0.00017	0.000345

The top 20 results obtained in the multi-run orp method are analyzed in each cell. Grand average (Ave) and SE are listed in the bottom rows.

### Complementary relationship among ***I***_***K1***_***, I***_***ha,***_ and ***I***_***bNSC***_

**Figure 5:**
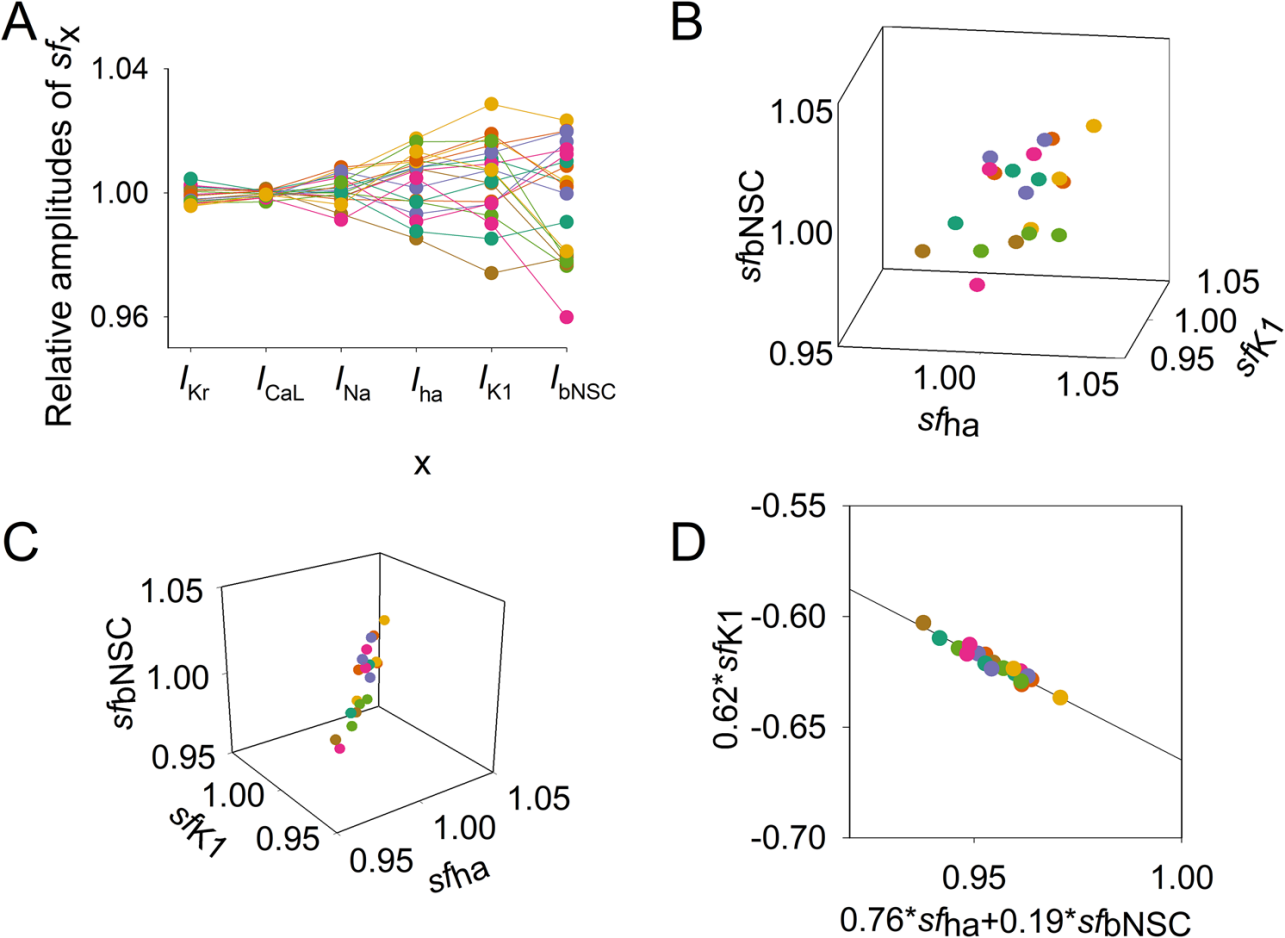
Distribution of *sf*_*x*_ in the top 20 sets of *sf*_*x*_s obtained from the multi-run orp test in Cell86 in Fig. 2. Data points of normalized *sf*_*x*_ in each set are depicted in different colors. (**A**) Plot of the amplitudes of each *sf*_*x*_ (indicated on the abscissa). (**B**) Three-dimensional plot of the three parameters of *sf*_*ha*_, *sf*_*K1,*_ and *sf*_*bNSC*_. (**C**) A different solid angle view of the three-dimensional plot showing a linear correlation; see text for the plot in (**D**).

Figure 5A illustrates the distribution of the *sf*_*x*_s amplitude in the top 20 data points. The final *sf*_*x*_s in individual runs were connected with lines for each run of PS in Cell 86 (Fig. 2). The SEM values were quite small (< 1%) in *sf*_*Kr*_ and *sf*_*CaL*_. In contrast, *sf*_*ha*_, *sf*_*K1*_, and *sf*_*bNSC*_ showed larger deviations. This finding is interesting because the former currents are mainly involved in determining the AP configuration and the latter group mainly drives the relatively long-lasting slow diastolic depolarization (SDD) of approximately 1 s duration.

Thus, we analyzed the distribution of *sf*_*ha*_, *sf*_*K1*_, and *sf*_*bNSC*_ in the top 20 MSE. Figure 5B,C show the distribution of the *sf*_*x*_ points in the space of the three *sf*_*x*_ dimensions. In Fig. 5B, the 20 data points seemed to be dispersed randomly in the parameter space. However, when the space was rotated to a specific angle, a linear distribution was observed (Fig. 5C), indicating that the points were distributed approximately on a plane surface in the three-dimensional space. Using multiple regression analysis, we obtained an equation that fit the 20 data points as follows (R^2^ = 0.872):

16 $$ \begin{array}{*{20}c} {0.762 \cdot sf_{ha} - 0.619 \cdot sf_{K1} + 0.191 \cdot sf_{bNSC} = 0.333554} \\ \end{array} $$

By replotting the data points in the two-dimensional space with the abscissa for the sum of two inward-going currents (0.76 *sf*_*ha*_ + 0.19 *sf*_*bNSC*_) and the ordinate for the outward current 0.62 *sf*_*K1*_, we obtained the regression line shown in Fig. 5D. Close correlations among the three *sf*_*x*_s were indicated with a high R^2^ of 0.941. This finding confirms that the three currents have complementary relationships with each other to provide virtually identical configurations of spontaneous AP. In other words, $log\left(MSE\right)$ remains nearly constant as long as the composition of the currents satisfies the relationship given by Eq. 16.

**Figure 6:**
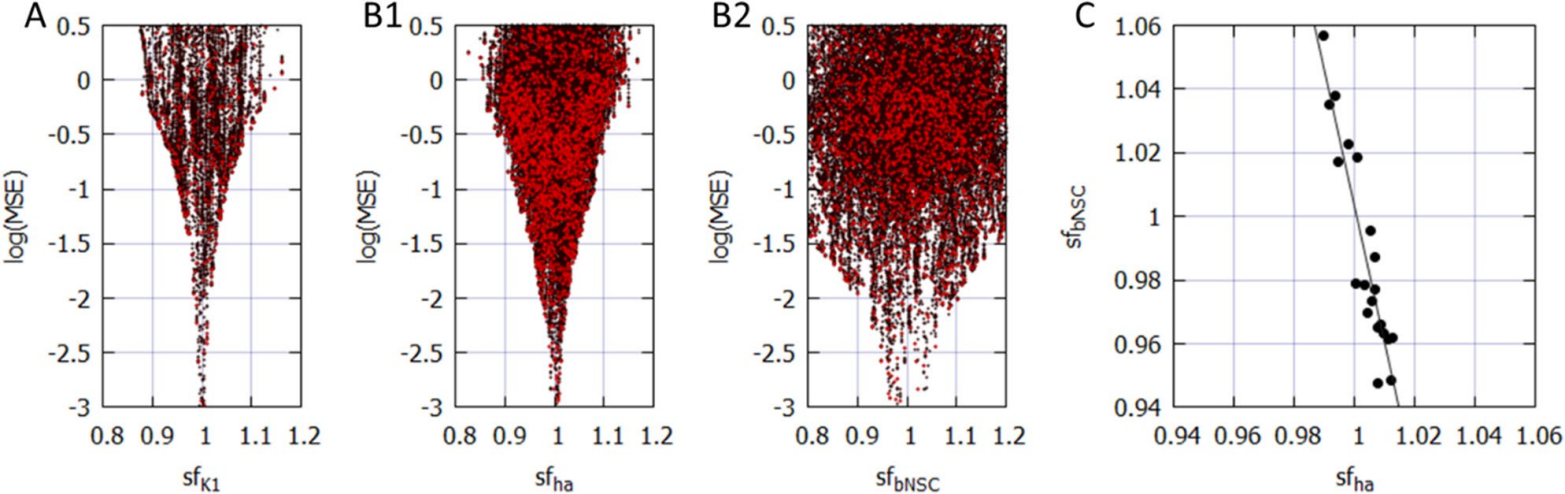
The complementary relations among *sf*_*K1*_, *sf*_*ha*_ and *sf*_*bNSC*_*.* (**A**) and (B) results of the multi-run orp test. A; the perfect convergence of *sf*_*K1*_ when *sf*_*ha*_ and *sf*_*bNSC*_ were fixed. (**B1**) improved convergence of *sf*_*ha*_ and (**B2**) *sf*_*bNSC*_ when *sf*_*K1*_ was fixed. In these two orp tests, *sf*_*x*_ of other currents showed quite comparable convergence as in Fig. 4A. (**C**) the correlation between *sf*_*ha*_ and *sf*_*bNSC*_.

The complementary relationship was further examined by performing an orp test after fixing one of the two factors, *sf*_*K1*_ or (*sf*_*ha*_ + *sf*_*bNSC*_), as illustrated in Fig. 5B. Figure 6A shows the $log\left(MSE\right)$ vs. *sf*_*K1*_ relation when (*sf*_*ha*_ + *sf*_*bNSC*_) was fixed at the values obtained by the orp test. Indeed, the typical convergence of the *sf*_*K1*_ was obtained. Alternatively, if the *sf*_*K1*_ was fixed, the convergence was obviously improved for both *sf*_*ha*_ and *sf*_*bNSC*_ (Fig. 6B-1,2), but it was less sharp if compared to *sf*_*Kr*_, *sf*_*CaL*_ and *sf*_*Na*_ (not shown, but refer to corresponding results in Fig. 4A). This finding was further explained by plotting the relationship between the two inward currents, *I*_*ha*_ and *I*_*bNSC,*_ as illustrated in Fig. 6C. The regression line for the data points was fitted by Eq. 17 with R^2^ = 0.86, supporting the complemental relationship between the two inward currents, *I*_*ha*_ and *I*_*bNSC*_.

17 $$ \begin{array}{*{20}c} {0.9736 \cdot sf_{bNSC} + 0.2281 \cdot sf_{ha} = 1.2024} \\ \end{array} $$

The moderately high R^2^ indicates that the SDD is determined not only by the major *I*_*ha*_ and *I*_*bNSC*_* but* also by other currents, such as *I*_*K1*_, *I*_*Kr*_, the delayed component of *I*_*Na*_ (*I*_*NaL*_*)* and *I*_*CaL*_, which were recorded during the SDD as demonstrated in Fig. 3.

Essentially the same results of complementary relationship among *sf*_*ha*_, *sf*_*bNSC*_ and *sf*_*K1*_ were obtained in Cell 91, which also showed the long-lasting SDD with the very negative MDP as in Cell 86, as shown in Fig. 2 and Table 2. The regression relation for the data points was fitted by Eqs. (18) and (19) with R^2^ = 0.656 and 0.472, respectively.

18 $$ \begin{array}{*{20}c} {0.572 \cdot sf_{ha} - 0.132 \cdot sf_{K1} + 0.810 \cdot sf_{bNSC} = 1.25891} \\ \end{array} $$

19 $$ \begin{array}{*{20}c} {0.9279 \cdot sf_{ha} + 0.3706 \cdot sf_{bNSC} = 1.30025} \\ \end{array} $$

### Principal components in the hiPSC-CM model

The PS frequently got stuck during the progress of parameter optimization and failed to reach the global minimum in the present study (Figs. 4,6). The major cause of this interruption may most probably be attributed to the fact that *sf*_*x*_s were used directly as the search vector of the PS. In principle, the algorithm of PS parameter optimization gives the best performance when the parameters search is conducted in orthogonal dimensions where each dimension does not affect the adjustment of other *sf*_*x*_^41^. To get deeper insights, we applied the principal component (PC) analysis to the set of 6 *sf*_*x*_s selected in the baseline model. We performed PC analysis on the data points recorded in the vicinity of the minima (using the top 20 data).

**Figure 7:**
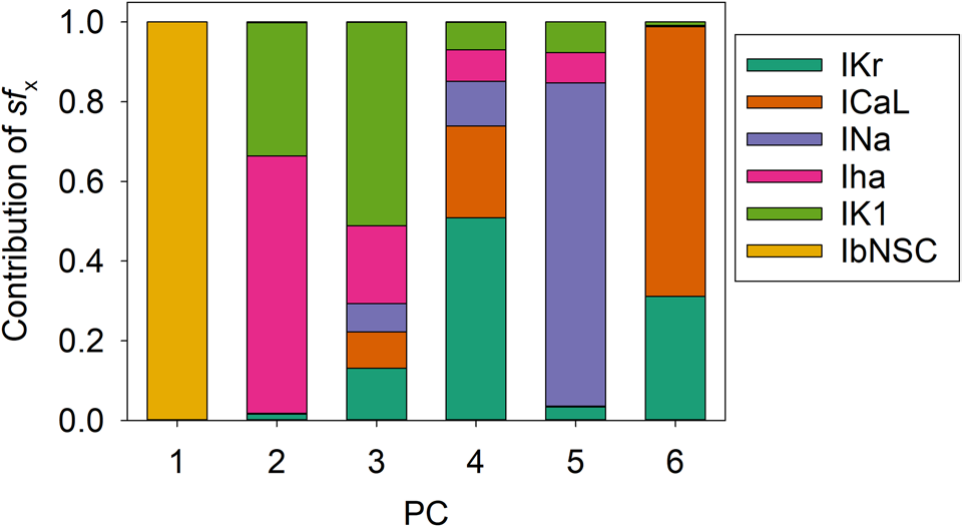
PC1 ~ 6 to describe distribution of the 6 *sf*_*x*_s. PC analysis was performed on the data population of the top 200 runs of the orp test as in Fig. 4, which showed good optimization results (Cell 86). Each magnitude of 6 PCs was normalized to give a unit magnitude. Note each PC is composed of multiple components of ionic current, which are indicated in the Index with corresponding colors.

As illustrated in Fig. 7, each of the 6 PCs was not composed of a single *sf*_*x*_ but mostly included multiple *sf*_*x*_ sub-components. This finding indicates the inter-parameter interactions during the process of parameter optimization. For example, the changes in *sf*_*K1*_ or *sf*_*bNSC*_ simultaneously affect PCNo.1, 3, 6 or 1, 2, 3 PCs, respectively. Both *sf*_*CaL*_ and *sf*_*Kr*_ affect PCNo.4, 5. It might be concluded that the frequent interruptions of PS parameter optimization are most probably caused by the sporadic appearance of the local minima of MSE through interactions among *sf*_*x*_s.

## Discussion

New findings in the present study are listed below.

1. Mapping of the MSE distribution over the enlarged parameter space was conducted by randomizing the *G*_*x*_s of the baseline model. It was confirmed that the baseline model had only a single sharp depression in MSE at the default *G*_*x*_s (Fig. 1).
2. The preliminary cell-specific models were firstly prepared by the conventional manual tuning of *G*_*x*_s to superimpose the model output on each of the 12 experimental AP recordings (Fig. 2). The parameter search space was restricted to a relatively small space to facilitate parameter optimization.
3. The *sf*_*x*_s of the 4–6 *G*_*x*_ parameters were initially assigned random values from a uniform distribution ranging between ± 10% of the default values. The MSE was calculated between the randomized model output and the intact model AP as the target of optimization (Fig. 3).
4. Plotting the parameter *sf*_*x*_ in common *sfx-MSE* coordinates during each run of several hundred runs of optimization (Fig. 4), revealed that the *sf*_*x*_ distributions of *I*_*Kr*_, *I*_*CaL*_, and *I*_*Na*_ converged sharply to a single point with decreasing MSE, which exactly equaled the default values. In contrast, estimates of *sf*_*K1*_, *sf*_*ha*_, and *sf*_*bNSC*_ deviated slightly within a limited range around the default values in cells showing long-lasting SDD (Fig. 4).
5. For statistical evaluation, the mean ± SE of *sf*_*x*_ in the top 20 MSE estimates was calculated for individual cells (Table 2). The results of the parameter optimization in the 12 cells indicated that the means of *sf*_*x*_s were very close to 1.00, with an SE < 0.01 for all *G*_*x*_*s*.
6. A complementary relationship was found between *sf*_*K1*_, *sf*_*ha*_, and *sf*_*bNSC*_ in determining the gentle slope of the long-lasting SDD in two representative cells (Fig. 5). Supporting this view, *sf*_*K1*_ clearly focused on the unit provided that *sf*_*ha*_ and *sf*_*bNSC*_ were fixed and vice versa (Fig. 6).
7. The six search vectors of *sf*_*x*_ in the presented model could be replaced by the same number of theoretical PCs, and each PC was mostly composed of multiple *sf*_*x*_*s* (Fig. 7). This finding supports the view^12^ that the complex interactions among *I*_*x*_s might interrupt the progress of the parameter optimization when *sf*_*x*_s are used as the search vector instead of using theoretical orthogonal ones.

The use of an initial randomized set of parameters was crucial in examining whether an optimization method could determine unique estimates independent of the initial set of parameters, as used in the GA-based method for determining the *G*_*x*_s of the mathematical cardiac cell model^23^. The aforementioned seven findings confirm the feasibility of the PS method. Most likely, the PS method is applicable to variable mathematical models of other cell functions. Reference^26^ provides a more systematic review of parameter optimization in cardiac model development.

It has been suggested that different combinations of parameters generate similar outputs^12,23–25^. In the present study, this suggestion was explained at least in part by the complementary relationship, for example, between *I*_*K1*_, *I*_*ha*_, and *I*_*bNSC*_ in determining d*V*_*m*_/dt of SDD, which is a function of the total current (Eq. 2, Figs. 5,6). The gradient-based optimization method relies on the precise variation in the time course of d*V*_*m*_/dt induced by time-dependent changes in individual *sf*_*x*_s (Eq. 2). Therefore, MSE was calculated over the entire time course of spontaneous APs. Notably, we did not use AP metrics, which indirectly reflect the kinetic properties of the individual currents. Even with this measure of calculating the MSE, the time-dependent changes in $pO$ (Eq. 3) might be relatively small between two major currents, *I*_*K1*_ and *I*_*ha*_*,* in comparison to *I*_*bNSC*_, which has no *V*_*m*_-dependent gate during the SDD, as shown in the current profile in Fig. 3A-3. We assume that the gradient-based optimization method can determine different contributions of individual currents if the optimization is conducted only within a selected time window of SDD. If the MSE is calculated over multiple phases of spontaneous AP, the influence of a particular phase on the MSE should be diluted. In our preliminary parameter optimization, this problem was partly solved using a weighted sum for different phases of the spontaneous AP in summing the MSE.

The small amplitude of a given current might be an additional factor in the weak convergence of *sf*_*x*_ observed in the diagram of *sf*_*x*_—MSE in the orp test of optimization. If the current amplitude is much smaller than the sum of all currents in determining d*V*_*m*_/dt (Eq. 2), the resolution of the PS method would decrease. Sarkar et al.^24^ demonstrated that the model output, for example, the AP plateau phase, was almost superimposable when different ratios of *G*_*Kr*_ and *G*_*pK*_ were used to reconstruct the model output (their Fig. 1). The authors reported that the AP metrics used for comparisons, such as APD, OS, and APA, were quite similar. However, these results were obtained by applying different combinations of *sf*_*x*_ to the same Ten Tusscher–Noble–Noble–Panfilov (TNNP) model^33^. This means that the relative amplitudes of *I*_*Kr*_ and *I*_*pK*_ in the TNNP model were much smaller than those of the major *I*_*CaL*_ during the AP plateau, even though *I*_*Kr*_ and *I*_*pK*_ have completely different gating kinetics. Thus, the results of the parameter optimization should be model-dependent. The same arguments can also be applied to the use of the FR guinea pig model^42^ in the study by Groenendaal et al.^23^.

A gradient-based parameter optimization method was applied to the cardiac model of membrane excitation in a study^12^ that analyzed the classic BR model^40^. The whole-cell current in the BR model is composed of a minimum number of ionic currents, background *I*_*K1*_, and three time-dependent currents (*I*_*Na*_, *I*_*s*_, and *I*_*x1*_) which were dissected from the voltage clamp experiments by applying the sucrose gap method to the multicellular preparation of ventricular tissue. The gating kinetics of the latter three currents were formulated according to Hodgkin-Huxley type gating kinetics, which is quite simple compared to the recent detailed description of ionic currents. The authors reported that the parameter optimization was difficult if the AP configuration was used as the target of the parameter optimization. They used the time course of the whole-cell current as a target for parameter optimization. However, the number of parameters was quite large (in their study, 63) and included limiting conductances and gating kinetics. The authors suggested that the feasibility of the parameter optimization method would be improved with additional experimental data.

In modern mathematical cardiac cell models, most ionic currents are identified by whole-cell voltage clamp and single-channel recordings in dissociated single myocytes^43^ using the patch-clamp technique^44^ and by identifying the molecular basis of membrane proteins. The molecular basis of the ion channels expressed in the hiPSC-CMs from the GSE154580 GEO Accession viewer is mostly identical to those in the adult cardiac myocytes, rather than in the fetal heart. Moreover, gating kinetics have been extensively studied to characterize ionic currents within the cell model. In principle, the detailed characterization of individual currents should facilitate the identifiability of the model parameter, but should not necessarily interfere with parameter optimization. We consider that the manual fitting of the model parameters to the AP recording using a priori knowledge of biophysical mechanisms should largely facilitate the subsequent automatic parameter optimization. Also, ionic currents left at the default values work as constraints to improve the identifiability of the target parameters.

After validating the automatic parameter optimization method, the final goal of our study was to determine the principle of ionic mechanisms that are applicable to the full range of variations in spontaneous AP records in both hiPSC-CMs and mature cardiomyocytes. The multi-run PS method was applied to the experimental AP recordings using the initial parameter sets obtained by the conventional manual fit. The protocol for measuring *G*_*x*_s was the same as that used in the present study, except for the use of experimental AP recordings instead of the output of the 'cell-specific model'. In our preliminary analysis, the magnitude of the individual model parameters obtained by manual tuning was corrected by < 15% by objective parameter optimization.

Finally, the ionic mechanisms underlying the SDD of variable time courses will be analyzed in a quantitative manner, for example, by using lead potential analysis^45^, which explains changes in *V*_*m*_ in terms of the *G*_*x*_ of individual currents. An example of applying the new PO method to the experimental recording of selective *I*_Kr_-blockade, yet still preliminary is described in four hiPSC-CMs (see Figs. S6 and S7 in Supplemental Materials).

## Limitations

There are several limitations in the present study. In general, the obvious limitations of published mathematical models of cardiac membrane excitation are caused by a shortage of functional components inherent in intact cells. For example, [ATP]_i_ that is controlled by energy metabolism is a vital factor in maintaining the physiological function of ion channels as well as the active transport of the Na^+^/K^+^ pump^46^. Moreover, most models do not account for modulation of the ion channel activity through phosphorylation of the channel proteins, detailed modulation of the channel by [Ca^2+^]_i_, alterations in ion channel activity by PIP_2_^47,48^, and tension of the cell membrane through changes in cell volume^49–52^. The detailed Ca^2+^ dynamics of [Ca^2+^]_i_ are still not implemented in most cardiac cell models. These dynamics include Ca^2+^ release from sarcoplasmic reticulum (SR) activated through the coupling of a few L-type Ca^2+^ channels with a cluster of ryanodine receptors (RyRs) at the dyadic junction^29^ and Ca^2+^ diffusion influenced by the Ca^2+^-binding proteins^53^. To simulate Ca^2+^ binding to troponin during the development of contraction, a dynamic model of the contracting fibers is necessary^54–57^. These limitations should be thoroughly considered when investigating pathophysiological phenomena such as arrhythmogenesis. The scope of the present study was limited to the AP configurations of hiPSC-CMs, which were assumed to be ‘healthy’ with respect to the above concerns. For example, [ATP]_i_, [Na^+^]_i_, and Ca_tot_ were kept constant, and the standard contraction model was implemented, as in the hVC model.

The parameter optimization presented in this study can be achieved in a practical manner by limiting the number of unknown parameters. We determined only *G*_*x*_s based on the assumption that ion channel kinetics are preserved, as in hiPSC-CMs and mature myocytes. Usually, four to six ionic currents are selected for optimization. The orp method could be performed simultaneously for all nine ionic currents, as described in Eq. (1). However, the computation time was radically prolonged and the resolution was not as high as that obtained using a modest number of parameters. We consider that the determination of a limited number of *G*_*x*_s is relevant to solving physiological problems in terms of detailed model equations for each current system.

Although *I*_*NCX*_ and *I*_*NaK*_ contributed sizeable fractions of the whole-cell outward and inward currents, respectively (Fig. 3A-3), we excluded the scaling factors *sf*_*NaK*_ and *sf*_*NCX*_ from the parameter optimization for the sake of simplicity. Instead, the possible drift of intracellular ion concentrations was fixed during the repetitive adjustment of ionic fluxes by varying *sf*_*x*_, as shown in Table 2. The introduction of the empirical equations (Eq. 13 and 14) was useful for adjusting [Na^+^]_i_ and Ca_tot_ (Table 2) so that the time course and magnitude of *I*_*NCX*_ remained almost constant during the parameter optimization. In future studies, when the influences of varying [Na^+^]_i_ and/or Ca_tot_ are examined under various experimental conditions, the reference levels of [Na^+^]_i_ and/or Ca_tot_ (*stdNai* and *stdCatot* in Eq. 13 and 14) might be replaced by experimental measurements.

For the excitation–contraction coupling and calcium-induced calcium release (CICR) in hiPSC-CMs lacking T-tubules, Koivumaki et al.^58^ developed the novel Paci model of hiPSC-CM with essential features of membrane electrophysiology and intracellular CICR with the spontaneous membrane excitation (mouse fetal cell model^58^) as a platform that can be used to facilitate the translational research from hiPSC-CMs to heart diseases^59^. This composite model demonstrated spontaneous Ca^2+^ release, which occurred several tens of milliseconds before the AP, to serve as a trigger. This is different from the almost simultaneous rise in spontaneous AP and the accompanying Ca^2+^ transient, as demonstrated by Spencer et al.^60^. These differences might most probably be due to the variable degrees of maturation of hiPSC-CMs used in different laboratories.

The issue of coupling CICR with cardiac membrane excitation in the absence of T-tubules has long been extensively discussed in sinoatrial (SA) node pacemaker cells. Maltsev and Lakatta proposed cell models in which APs were triggered by the gradual increase in [Ca^2+^] in the heuristic submembrane space during the Ca^2+^-transient (the 'Ca-clock' theory)^61,62^. Himeno et al. examined this issue using patch-clamp experiments in isolated SA node pacemaker cells^63^. The authors described that the spontaneous rhythm remained intact when SR Ca^2+^ dynamics were acutely disrupted by addition of high doses of a Ca^2+^-chelating agent to the cytosol. This experimental finding could be reconstructed using their SA node cell model, supporting the membrane origin of spontaneous AP generation. A more detailed and extensive theoretical study was published by Stern et al.^64^ (see also Hinch et al.^4^). The authors constructed a computational cell model that included the three-dimensional diffusion and buffering of Ca^2+^ in the cytosol. The Ca^2+^-releasing couplon was located at the site of close contact of the junctional SR membrane with the cell membrane, where the individual clusters of RyRs of various sizes on the SR membrane and a few LCC on the cell membrane are functionally coupled across the nanoscale gap. Interestingly, no local Ca^2+^ release occurred if the clusters of RyRs were separated by > 1 μm. However, bridging large RyR clusters to form an irregular network can lead to the generation of propagating local CICR events and partial periodicity, as observed experimentally. Considering all these experimental and theoretical findings, the issue of ‘Ca-clock’ is still a matter of debate. Therefore, we consider that including all these details in the hiPSC version of the hVC model is clearly beyond the scope of the present study, which aims to develop a PO method to determine the parameters of the membrane excitation model in general.

The PO method was not applied to several ionic currents in this study. For example, it was difficult to determine the kinetics of the T-type Ca^2+^ channel (*I*_*CaT*_; Ca_V_ 3.1) and so it was excluded from the present study. The very fast opening and inactivation rates that have been previously described^65^ suggest a complete inactivation of *I*_*CaT*_ over the voltage range of SDD, while the sizeable magnitude of the window current that has also been described^66^ suggests a much larger contribution to SDD. The kinetics of *I*_*CaT*_ remain to be clarified through experimental examination. The sustained inward current, *I*_*st*_, has recently been attributed most probably to Ca_V_ 1.3^67^, which is activated at a more negative potential range than *I*_*CaL*_ (Ca_V_ 1.2)^68,69^. In the present study, *I*_*bNSC*_ was used to represent the net background conductance. However, several components of background conductance have been identified at the molecular level in mature myocytes (for a review of TRPM4, see^70^). Experimental measurements of the current magnitude of each component are required.

Gábor and Banga indicated that the multi-run method performed well in certain cases, especially when high-quality first-order information was used, and the parameter search space was restricted to a relatively small domain^16^. Another study echoed these findings^19^. In the present study, manual fitting of the parameters (Fig. 1) was required to utilize the multi-run PS method over the restricted search space. One of the major difficulties in the manual fitting of individual *G*_*x*_s arose during SDD, where *I*_*Kr*_, *I*_*K1*_, *I*_*bNSC*_, and *I*_*ha*_, in addition to *I*_*NaK*_ and *I*_*NCX*_, constitute the whole-cell current (Fig. 3A-3). However, close inspection of the current components in Fig. 3A-3 provides hints on how to do with the manual fit. The transient peak of *I*_*Kr*_ dominates the current profile during the final repolarization phase from -20 to -60 mV in all 12 hiPSC-CMs^71^, since *I*_*CaL*_ and *I*_*Ks*_ rapidly deactivates before repolarizing to this voltage range. *I*_*NaK*_ and *I*_*NCX*_ are well controlled by the extrinsic regulation in Eqs. (13) and (14). Thus, manual fitting of *sf*_*Kr*_ was first applied to determine *sf*_*Kr*_. The MDP more negative than -70 mV was adjusted by the sum of time-dependent (*I*_*Kr*_ + *I*_*K1*_) and time-independent *I*_*bNSC*_. Then, *I*_*Kr*_ is deactivated when depolarization becomes obvious after the MDP, and the depolarization-dependent blocking of *I*_*K1*_ by intracellular substances^72^ play major roles in promoting the initial linear phase of SDD. Thus, the amplitudes of *sf*_*K1*_ and *sf*_*bNSC*_ may be approximated during the initial half of the SDD. The latter half of SDD, including the foot of AP (i.e., the exponential time course of depolarization toward the rapidly rising phase of AP) was mainly determined by the subthreshold *V*_*m*_-dependent activation of *I*_*Na*_ (after MDP more negative than -70 mV) and/or *I*_*CaL*_ (after MDP less negative than -65 mV). Thus, *sf*_*Na*_ and *sf*_*CaL*_ were roughly determined by fitting the foot of the AP and the timing of the rapid rising phase of AP. The plateau time course of AP is determined by *sf*_*CaL*_ and the Ca^2+^-mediated inactivation of *I*_*CaL*_ (parameter *KL*^4^). Because the kinetics of outward currents *I*_*Kur*_, *I*_*Kto*_ (endo-type), and *I*_*Ks*_ are quite different from those of *I*_*Kr*_, the plateau configuration was determined incrementally by adjusting these currents. We failed to observe phase 1 rapid and transient repolarization in hiPSC-CMs (Fig. 2), which is a typical sign of the absence of epicardial-type *I*_*Kto*_.

In hiPSC-CMs showing less negative MDP than approximately -65 mV, the contribution of *I*_*K1*_, *I*_*Na*_, and *I*_*ha*_ should be negligibly small because *I*_*K1*_ is nearly completely blocked by intracellular Mg^2+^ and polyamines, *I*_*Na*_ is inactivated, and *I*_*ha*_ is deactivated during SDD, even if it is expressed.

Nevertheless, parameter optimization might be laborious and time-consuming for those unfamiliar with the electrophysiology of cardiac myocytes. This difficulty might be largely eased by accumulating both AP configurations and the underlying current profile obtained in parameter optimization into a database in the future. If this database becomes available, computational searches for several candidate APs for the initial parameter set will be feasible, which will be used for automatic parameter optimization.

## Supplementary Information

Supplementary Information.

## Data Availability

The AP records used in Section 4.2, and the source code of the optimization program are available in the Supplemental Material link for the following bioRxiv entry. https://doi.org/10.1101/2022.05.16.492203.

## Abbreviations

hiPSC-CMs: Human induced pluripotent stem cell-derived cardiomyocytes
hVC model: The human ventricular cell model
AP: Action potential
MDP: The maximum diastolic potential
SDD: Slow diastolic depolarization
*I*_m_: Membrane current
*V*_m_: Membrane voltage
orp: Optimization of randomized model parameters
OS: Overshoot potential
PO method: Parameter optimization method
PS method: Pattern search method
BP: Base point for searching minimum MSE in the Pattern Search
NP: Searching point in reference to BP in the Pattern Search
MSE: Mean square error between two different *V*_*m*_ records
Stp: Step size to move NP
x: Subscript to represent membrane current, such as *I*_*Na*_*, I*_*CaL*_*, I*_*K1,*_* I*_*ha*_*, I*_*Kr*_*, I*_*Kur*_*, I*_*Ks*_*,* and *I*_*bNSC*_
