# Supplementary material for: Gradient-based parameter optimization method to determine membrane ionic current composition in human induced pluripotent stem cell-derived cardiomyocytes
Source: Sci Rep. 2022 Nov 9;12:19110. doi: 10.1038/s41598-022-23398-0 (PMC9646722; doi:10.1038/s41598-022-23398-0)
Supplement: Supplementary file 1 — Supplementary Information. [file 41598_2022_23398_MOESM1_ESM.pdf]

Supplemental Materials

Abbreviations

Table S1. Abbreviations in model equations

V_m	membrane potential (mV)
I_{tot_cell}	total current of ion channels and exchangers (pA/pF)
$I_{tot\_x\_a}$	total current of ion 'x' channels and exchangers at space 'a' (pA/pF)
I_{app}	current applied through a patch electrode (pA/pF)
$E_{rev,x}$	reversal potential of current 'x' (mV) determined from the tangential line of the I_x - V curve
C_m	whole cell membrane capacitance (pF)
G_x	conductance of current 'x' (pA /pF/mV)
k, α, β	rate constants (ms^{-1} or $mM^{-1}ms^{-1}$)
$P_{I(\_X)}$	converting factor of GHK_X from mM/ms to pA/mM/pF
v_{cyc_x}	turnover rate of transporter 'x' (ms^{-1})
V_a	total volume of space 'a' (fL)
$[X_{tot}]_a$	total concentration of substance 'X' at space 'a' (mM)
$[X_{free}]_a$	concentration of free substance 'X' at space 'a' (mM)
$[X]_a$	concentration of 'X' at space 'a' (mM)
J_X	total flux of ion 'X' (attomol/ms)
$\frac{d[X]_a}{dt}$	rate of change of 'X' concentration at space 'a' (mM/ms)

Model Parameters

Table S2. Physical constants

R	8.3143	C·mV/mmol/K
T	310.15	K
F	96.4867	C/mmol

Table S3. Ion concentrations

$[K^+]_o$	5.4	mM
$[Na^+]_o$	145	mM
$[Ca^{2+}]_o$	1.8	mM

GHK equation

The magnitudes of ion channel currents are described either by the Ohmic equation or by the GHK equation. In the latter case, the term to convert mM to pA (permeability times, *zF*) in the original GHK equation is represented by a lumped converting factor, P in a unit of pA/pF mM^{-1}, because of unknown total number of channels within a cell and single channel conductance. Then, the amplitude of current (I) for an ion X is given by,

$$I = P \cdot pO \cdot GHK_X \qquad Eq.S1$$

Where GHK_X is,

$$GHK_X = \frac{Z_X F V_m}{RT} \cdot \frac{\left([X]_i - [X]_o \cdot exp\left(\frac{-Z_X F V_m}{RT}\right)\right)}{\left(1 - exp\left(\frac{-Z_X F V_m}{RT}\right)\right)} \qquad Eq.S2$$

Nernst equation

$$E_X = \frac{RT}{z_X F} \cdot ln\left(\frac{[X]_o}{[X]_i}\right) \qquad Eq.S3$$

Table S4. Volume composition of cytosol in comparison with hVC model (Asakura et al., 2014, Himeno et al., 2015)

	hiPSC-CM	hVC model
Input capacitance	32 pF	192.46 pF
Cell volume (V_{cell})	2510 fL	37920 fL
Bulk space (V_{blk})	65% of V_{cell} fL	68% of V_{cell} fL
Intermediate zone (V_{iz})	3.5% of V_{cell} fL	3.5% of V_{cell} fL
Junctional space (V_{jnc})	0.8% of V_{cell} fL	0.8% of V_{cell} fL
Total SR space (V_{SR})	1.1% of V_{cell} = 27.61 fL	6% of V_{cell} fL
Volume of SR releasing site (V_{SRrl})	10% of V_{SR} fL	20% of V_{SR} fL
Volume of SR releasing site (V_{SRup})	90% of V_{SR} fL	80% of V_{SR} fL

Ca^{2+} buffer

The detailed set of buffer species used in the GPB model (2010) (Grandi et al., 2010) was adopted after several simplifications as described in our previous paper (Asakura et al., 2014). In short, we deleted the myosin, Na^{+} and Mg^{2+} buffers, and fixed $[Mg^{2+}]$. The low affinity binding of Ca^{2+} to troponin (TnCl) was replaced by a contraction model (Negroni and Lascano, 2008) and the amount of the high affinity site (TnCh) was adjusted.

Bulk space (blk)

$$\frac{d[CaMCa]}{dt} = k_{on\_CaM} \cdot [Ca^{2+}]_{blk} \cdot ([B_{total}CaM] - [CaMCa]) - k_{off\_CaM} \cdot [CaMCa] \quad Eq.S4$$

$$k_{off\_CaM} = 0.0238, k_{on\_CaM} = 3.4, [CaM_{tot}] = 0.024 \quad Eq.S5$$

$$\frac{d[TnChCa]}{dt} = k_{on\_TnCh} \cdot [Ca^{2+}]_{blk} \cdot ([B_{tot}TnCh] - [TnChCa]) - k_{off\_TnCh} \cdot [TnChCa] \quad Eq.S6$$

$$k_{off\_TnCh} = 0.000032, k_{on\_TnCh} = 2.37, [TnCh_{tot}] = 0.007 \quad Eq.S7$$

$$\frac{d[SRCa]}{dt} = k_{on\_SR} \cdot [Ca^{2+}]_{blk} \cdot ([B_{tot}SR] - [SRCa]) - k_{off\_SR} \cdot [SRCa] \quad Eq.S8$$

$$k_{off\_SR} = 0.006, k_{on\_SR} = 10, [SR] = 0.0171 \quad Eq.S9$$

Intermediate zone (iz)

$$[L_{free}]_{iz} = \frac{[B_{tot}L]_{iz}}{1 + \frac{[Ca^{2+}]_{iz}}{K_{dL\_iz}}}, [B_{tot}L] = 0.6078 \quad Eq.S10$$

$$K_{dL\_iz} = \frac{k_{off\_L\_iz}}{k_{on\_L\_iz}}, k_{off\_L\_iz} = 1.3, k_{on\_L\_iz} = 100 \quad Eq.S11$$

$$[H_{free}]_{iz} = \frac{[B_{tot}H]_{iz}}{1 + \frac{[Ca^{2+}]_{iz}}{K_{dH\_iz}}}, [B_{tot}H] = 0.2178 \qquad Eq. S12$$

$$K_{dH\_iz} = \frac{k_{off\_H\_iz}}{k_{on\_H\_iz}}, k_{off\_H\_iz} = 0.03, k_{on\_H\_iz} = 100 \qquad Eq. S13$$

$$[Ca^{2+}]_{iz} = \frac{[Ca_{tot}]_{iz}}{1 + \frac{[Lf]_{iz}}{K_{dL\_iz}} + \frac{[Hf]_{iz}}{K_{dH}}} \qquad Eq. S14$$

Junctional space (jnc)

$$[L_{free}]_{jnc} = \frac{[B_{tot}L]_{jnc}}{1 + \frac{[Ca^{2+}]_{jnc}}{K_{dL\_jnc}}}, [B_{tot}L] = 1.1095 \qquad Eq. S15$$

$$K_{dL\_jnc} = \frac{k_{off\_L\_jnc}}{k_{on\_L\_jnc}}, k_{off\_L\_jnc} = 1.3, k_{on\_L\_jnc} = 100 \qquad Eq. S16$$

$$[H_{free}]_{jnc} = \frac{[B_{tot}H]_{jnc}}{1 + \frac{[Ca^{2+}]_{jnc}}{K_{dH\_jnc}}}, [B_{tot}H] = 0.398 \qquad Eq. S17$$

$$K_{dH\_jnc} = \frac{k_{off\_H\_jnc}}{k_{on\_H\_jnc}}, k_{off\_H\_jnc} = 0.03, k_{on\_H\_jnc} = 100 \qquad Eq. S18$$

$$[Ca^{2+}]_{jnc} = \frac{[Ca_{tot}]_{jnc}}{1 + \frac{[Lf]_{jnc}}{K_{dL\_jnc}} + \frac{[Hf]_{jnc}}{K_{dH}}} \qquad Eq. S19$$

Release site of the SR (SRrl)

$$k_{off\_CSQN} = 65, k_{on\_CSQN} = 100, [B_{tot}CSQN] = 10 \qquad Eq. S20$$

$$K_{d\_CSQN\_Ca} = \frac{k_{off\_CSQN}}{k_{on\_CSQN}} \qquad Eq.S21$$

$$a = 1 \qquad Eq.S22$$

$$b = [B_{tot}CSQN] - \left[Ca_{tot}\right]_{SRrl} + K_{d\_CSQN\_Ca} \qquad Eq.S23$$

$$c = -K_{d\_CSQN\_Ca} \cdot \left[Ca_{tot}\right]_{SRrl} \qquad Eq.S24$$

$$[Ca^{2+}]_{SRrl} = \frac{-b + \sqrt{b^2 - 4ac}}{2a} \qquad Eq.S25$$

Boundary Ca^{2+} diffusion

Ca^{2+} transfer between cytosolic compartments

$$J_{Ca\_jnciz} = G_{dCa\_jnciz} \cdot \left([Ca^{2+}]_{jnc} - [Ca^{2+}]_{iz}\right) \qquad Eq.S26$$

$$G_{dCa\_jnciz} = 32158\ (fL \cdot ms^{-1}) \qquad Eq.S27$$

$$J_{Ca\_izblk} = G_{dCa\_izblk} \cdot ([Ca^{2+}]_{iz} - [Ca^{2+}]_{blk}) \qquad Eq.S28$$

$$G_{dCa\_izblk} = 2076.1\ (fL \cdot ms^{-1}) \qquad Eq.S29$$

Ca2+ transfer from SR uptake site to release site

$$J_{trans\_SR} = P_{trans} \cdot \left([Ca^{2+}]_{SRup} - [Ca^{2+}]_{SRrl}\right) \qquad Eq.S30$$

$$P_{trans} = 0.017(fL \cdot ms^{-1}) \qquad Eq.S31$$

Ion channels and transporters

L-type Ca^{2+} current (I_{CaL}, LCC)

According to the scheme of (Shirokov et al., 1993) and (Ferreira et al., 1997), the same 4-state model was used for both LCCs in CaRU (I_{CaL_jnc}) and for LCCs located in *blk* (I_{CaL_blk}) and *iz* (I_{CaL_iz}). The description of both V_m-dependent gate and [Ca^{2+}]-dependent gates in hVC model was used in the hiPSC-CM model after minor modification. The rate constants for the V_m-gate (α_+ and α_-) and Ca^{2+}-gate (ε_+ and ε_-) of LCC are

given by *Eqs. S35, S36* and *Eqs. S37, S38*, respectively. Both activation (α_+) and deactivation (α_-) rates of the V_m-gate were described as a function of two exponential terms and adjusted to hiPSC-CM data.

$$I_{CaL\_X\_a} = f_{CaL\_a} \cdot P_{CaL\_X} \cdot GHK_{X\_a} \cdot pO_{LCC\_a} \cdot \frac{1}{1 + \left(\frac{1.4}{[ATP]}\right)^3} \qquad Eq.S32$$

[ATP] was fixed to 6 mM.

Fraction of I_{CaL}

$$f_{CaL\_jnc} = 0.15,\ f_{CaL\_blk} = 0.45,\ f_{CaL\_iz} = 0.40 \qquad Eq.S33$$

Converting factors

$$P_{CaL\_Ca} = 5.068,\ P_{CaL\_Na} = 0.0000185 \cdot P_{CaL\_Ca},\ P_{CaL\_K} = 0.000367 \cdot P_{CaL\_Ca}\ (pA/pF/mM)$$

The rate constants for the V_m-gate,

$$v = V_m - V_{shiftCa} \qquad \mathrm{Eq.}S34$$

$$\alpha_+ = \frac{1}{0.763 \cdot exp\left(-\frac{v}{8.5}\right) + 0.348 \cdot exp\left(-\frac{v}{3500}\right)} \qquad Eq.S35$$

$$\alpha_- = \frac{0.5}{4.65 \cdot exp\left(\frac{v}{15}\right) + 1.363 \cdot exp\left(\frac{v}{100}\right)} \qquad Eq.S36$$

The rate constant (ε_+) for the Ca^{2+}-inactivation.

$$\varepsilon_+ = \frac{0.35 \cdot [Ca^{2+}]_{nd} \cdot \alpha_+}{T_L \cdot K_L} \qquad Eq.S37$$

The values of T_L (= 147.51) and K_L (=0.00396 mM) were determined by referring to the experimental measurements of steady-state inactivation. The rate of removing Ca^{2+} inactivation (ε_-) used in hVC model was used.

$$\varepsilon_- = \frac{1}{8084 \cdot exp\left(\frac{V_m}{10}\right) + 158 \cdot exp\left(\frac{V_m}{1000}\right)} + \frac{1}{134736 \cdot exp\left(-\frac{V_m}{5}\right) + 337 \cdot exp\left(-\frac{V_m}{2000}\right)} \qquad Eq.S38$$

The composition of whole cell I_{CaL}.

$$I_{CaL} = (I_{CaL\_Ca\_jnc} + I_{CaL\_Na\_jnc} + I_{CaL\_K\_jnc}) + (I_{CaL\_Ca\_iz} + I_{CaL\_Na\_iz} + I_{CaL\_K\_iz}) + (I_{CaL\_Ca\_blk} + I_{CaL\_Na\_blk} + I_{CaL\_K\_blk}) \quad Eq.S39$$

The sustained inward current (*Ist*)

In the spontaneous SA node cells, a sustained inward current was activated on depolarization to more negative potential range (V_m =~ -60 mV) than the usual threshold of the L-type Ca^{2+} current. The characteristics of the current was roughly similar to I_{CaL}, except that I_{st} was resistant to the removal of Ca^{2+} from the external solution and it was suggested that I_{st} is most probably carried by Na^+ (Guo et al., 1995, Mitsuiye et al., 1999). Recently, Toyoda et al. (2017) suggested that this current is generated by Cav.1.3, which is the major subtype expressed in the SA node cells. In the present study, we calculated I_{st} in the iPSC_CMs for convenience of comparing the role of I_{st} between the iPSC_CMs and the matured SA node cells. If appropriate, the sum of (I_{st} and the conventional I_{CaL}) was calculated.

$$I_{st} = I_{st,Na} + I_{st,K} \quad \text{Eq. S40}$$

$$I_{st,Na} = P_{stNa} \cdot GHK_{Na} \cdot pO \quad P_{stNa} = 0.00236\, pA/pF/mM \quad \text{Eq. S41}$$

$$I_{st,K} = P_{stK} \cdot GHK_K \cdot pO \quad P_{stK} = 0.585 \cdot P_{stNa}\, pA/pF/mM \quad \text{Eq. S42}$$

$$P_O = d \cdot f \cdot u \quad \text{Eq. S43}$$

$$(1-y) \underset{\beta}{\overset{\alpha}{\rightleftarrows}} y \qquad y = \{d, f, u\} \quad \text{Eq. S44}$$

$$\alpha_d = \frac{1}{0.15 \cdot Exp(\frac{V_m}{-11}) + 0.2 \cdot Exp(\frac{V_m}{-700})} \quad \text{Eq. S45}$$

$$\beta_d = \frac{1}{16 \cdot Exp(\frac{V_m}{8}) + 15 \cdot Exp(\frac{V_m}{50})} \quad \text{Eq. S46}$$

$$\alpha_f = \frac{1}{3100 \cdot Exp(\frac{V_m}{13}) + 700 \cdot Exp(\frac{V_m}{70})} \quad \text{Eq. S47}$$

$$\beta_f = \frac{1}{95 \cdot Exp(\frac{V_m}{-10}) + 50 \cdot Exp(\frac{V_m}{-700})} + \frac{2.5 \cdot [Ca^{2+}]_{blk}}{1 + Exp(\frac{V_m}{-5})} \qquad \text{Eq. S48}$$

$$\alpha_u = \frac{1}{400000 \cdot Exp(\frac{V_m}{9}) + 60 \cdot Exp(\frac{V_m}{65})} \qquad \text{Eq. S49}$$

$$\beta_u = \frac{1}{700 \cdot Exp(\frac{V_m}{-14}) + 60 \cdot Exp(\frac{V_m}{-65})} \qquad \text{Eq. S50}$$

T-type Ca^{2+} current (I_{CaT})

I_{CaT} is assumed in the *blk* space.

$$I_{CaT} = 2 \cdot P_{CaT} \cdot GHK_{Ca} \cdot pO_{CaT}, \ P_{CaT} = 9.56 \qquad Eq.S51$$

$$pO_{CaT} = d \cdot f \qquad Eq.S52$$

$$\alpha_d = \frac{1}{0.019 \cdot exp\left(-\frac{V_m}{5.6}\right) + 0.82 \cdot exp\left(-\frac{V_m}{250}\right)} \qquad Eq.S53$$

$$\beta_d = \frac{1}{40 \cdot exp\left(\frac{V_m}{6.3}\right) + 1.5 \cdot exp\left(\frac{V_m}{10000}\right)} \qquad Eq.S54$$

$$\alpha_f = \frac{1}{62000 \cdot exp\left(\frac{V_m}{10.1}\right) + 30 \cdot exp\left(\frac{V_m}{3000}\right)} \qquad Eq.S55$$

$$\beta_f = \frac{1}{0.0006 \cdot exp\left(-\frac{V_m}{6.7}\right) + 1.2 \cdot exp\left(-\frac{V_m}{25}\right)} \qquad Eq.S56$$

The hyperpolarization-activated current (I_{ha} or I_f)

In 1976, Noma and Irisawa for the first time conducted the double-microelectrode voltage clamp in a man-made small tissue preparation (0.2~0.3 mm in diameter) of the rabbit SA node tissue. They found a very slow activation time course of inward current (I_h) on hyperpolarization from the holding potential of -40 mV. Yanagihara and Irisawa (1980) clearly separated I_h from the delayed rectifier K current by the difference in the

activation range and the Ba^{2+}-resistant nature of I_{ha}. They measured the fully activated I-V relationship with the reversal potential is at -25 mV, suggesting little sensitivity to any particular ion species. They developed the Hodgkin-Huxley type kinetic model of I_{ha}, and suggested that I_{ha} plays a significant role in keeping the pacemaker cell at a low membrane potential, but only a small role in promoting the slow diastolic depolarization because of its time constant of several seconds. Yanagihara et al., (1980) published the mathematical model of the SA node cell action potential. The detailed I_{ha} model described by Maruoka et al., (1994) was used to reflect the delay in both activation and deactivation on hyper- and de-polarizations, respectively.

$$I_{ha} = I_{ha,Na} + I_{ha,K}$$ Eq. S57

$$I_{ha,Na} = P_{ha,Na} \cdot GHK_{Na} \cdot pO \qquad P_{ha,Na} = 0.03642 \quad pA/pF/mM$$ Eq. S58

$$I_{ha,K} = P_{ha,K} \cdot GHK_{K} \cdot pO \qquad P_{ha,K} = 4.244 \cdot P_{ha,Na} \quad pA/pF/mM$$

Eq. S59

$$C1 \underset{\lambda}{\overset{\mu}{\rightleftarrows}} C2 \underset{\beta}{\overset{\alpha}{\rightleftarrows}} O1 \underset{\beta}{\overset{\alpha}{\rightleftarrows}} O2 \underset{\beta}{\overset{\alpha}{\rightleftarrows}} O3$$ Eq. S60

$$\alpha_{ha} = \frac{1}{3500 \cdot \exp(\frac{V_m}{16.8}) + 0.3 \cdot \exp(\frac{V_m}{400})}$$ Eq. S61

$$\beta_{ha} = \frac{1}{4 \cdot \exp(\frac{V_m}{-14}) + 2 \cdot \exp(\frac{V_m}{-400})}$$ Eq. S62

$$\mu_{ha} = \frac{1}{45000000 \cdot \exp(\frac{V_m}{8.7}) + 500 \cdot \exp(\frac{V_m}{200})}$$ Eq. S63

$$\lambda_{ha} = \frac{1}{10.5 \cdot \exp(\frac{V_m}{-16.4}) + 0.4 \cdot \exp(\frac{V_m}{-400})}$$ Eq. S64

In the steady-state, the full model is reduced to a two-state transition model of a closed state (Ct) and an open state (Ot) to obtain the steady-state open probability (pOtSS)

$$Ct \underset{kb}{\overset{kf}{\rightleftarrows}} Ot \qquad \text{Eq. S65}$$

where,

$$kf = \frac{\alpha}{\frac{\lambda}{\mu}+1} \qquad kb = \frac{\beta}{1+\frac{\alpha}{\beta}+(\frac{\alpha}{\beta})^2} \qquad pOt_{SS} = \frac{kf}{kf+kb} \qquad \text{Eq. S66}$$

Sodium current (I_{Na})

The same I_{Na} model as in our previous study (Asakura et al., 2014) was used, except for the amplitude parameters, f_L and P_{Na}. I_{Na} is composed of the two components, I_{NaT} and I_{NaL}. The scheme for the state transition is shown below.

$$I_{Na} = I_{NaT} + I_{NaL} \qquad Eq.S67$$

$$f_L = \frac{I_{NaL}}{I_{NaT}+I_{NaL}} = 0.04\ or\ 0.01 \qquad Eq.S68$$

$$I_{NaT} = (1-f_L)\cdot P_{Na}\cdot(GHK_{Na}+0.18\cdot GHK_K)\cdot p(O)_{NaT} \qquad Eq.S69$$

$$I_{NaL} = f_L\cdot P_{Na}\cdot(GHK_{Na}+0.18\cdot GHK_K)\cdot p(O)_{NaL} \qquad Eq.S70$$

$$P_{Na\_Na} = 73.77825, P_{Na\_K} = 0.18\cdot P_{Na\_Na}\ (pA/pF/mM)$$

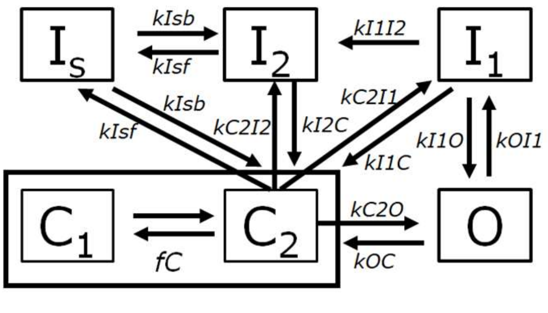

Transient component ($\boldsymbol{I_{NaT}}$)

$$\frac{dp(O)_{NaT}}{dt} = k_{I_2O} \cdot p(I_2)_{NaT} + f_{C\_Na} \cdot k_{C_2O} \cdot p(C)_{NaT} - \left(k_{OC} + k_{OI_2}\right) \cdot p(O)_{NaT} \qquad Eq.S71$$

$$\frac{dp(I_2)_{NaT}}{dt} = f_{C\_Na} \cdot k_{C_2I_2} \cdot p(C)_{NaT} + k_{OI_2} \cdot p(O)_{NaT}$$
$$+k_{I_sb} \cdot\ p(I_s)_{NaT} - \left(k_{I_2C} + k_{I_2O} + k_{I_sf}\right) \cdot p(I_2)_{NaT} \qquad Eq.S72$$

$$\frac{dp(I_s)_{NaT}}{dt} = k_{I_sf} \cdot\ p(I_2)_{NaT} + k_{I_sf} \cdot p(C)_{NaT} - 2 \cdot k_{I_sb} \cdot\ p(I_s)_{NaT} \qquad Eq.S73$$

$$p(C)_{NaT} = 1.0 - p(I_s)_{NaT} - p(O)_{NaT} - p(I_2)_{NaT} \qquad Eq.S74$$

$$f_{C\_Na} = \frac{C_2}{C_1 + C_2} = \frac{1}{1 + exp\left(-\frac{v + 48}{7}\right)} \qquad Eq.S75$$

$$v = Vm - VshiftNa \qquad \text{Eq.}S76$$

$$k_{C_2O} = \frac{1}{0.0025 \cdot exp\left(-\frac{v}{8.0}\right) + 0.15 \cdot exp\left(-\frac{v}{100.0}\right)} \qquad Eq.S77$$

$$k_{OC} = \frac{1}{30.0 \cdot exp\left(\frac{v}{12.0}\right) + 0.53 \cdot exp\left(\frac{v}{50.0}\right)} \qquad Eq.S78$$

$$k_{OI_2} = \frac{1}{0.0433 \cdot exp\left(-\frac{V_m}{27.0}\right) + 0.34 \cdot exp\left(-\frac{V_m}{2000.0}\right)} \qquad Eq.S79$$

$$k_{I_2O} = 0.0001312 \qquad Eq.S80$$

$$k_{C_2I_2} = \frac{0.5}{1.0 + \frac{k_{I_2O} \cdot k_{OC}}{k_{OI_2} \cdot k_{C_2O}}} \qquad Eq.S81$$

$$k_{I_sb} = \frac{1}{300000.0 \cdot exp\left(\frac{V_m}{10.0}\right) + 50000.0 \cdot exp\left(\frac{V_m}{16.0}\right)} \qquad Eq.S82$$

$$k_{I_sf} = \frac{1}{0.016 \cdot exp\left(-\frac{V_m}{9.9}\right) + 8.0 \cdot exp\left(-\frac{V_m}{45.0}\right)} \qquad Eq.S83$$

Late component (I_{NaL})

The $k_{I_1I_2}$, k_{OI_1}, k_{I_1O}, k_{I_1C} and $k_{C_2I_1}$ are specific for I_{NaL}, and other rate constants are the same as in I_{NaT}.

$$\frac{dp(O)_{NaL}}{dt} = k_{I_1O} \cdot p(I_1)_{NaL} + f_{C\_Na} \cdot k_{C_2O} \cdot p(C)_{NaL} - \left(k_{OC} + k_{OI_1}\right) \cdot p(O)_{NaL} \qquad Eq.S84$$

$$\frac{dp(I_1)_{NaL}}{dt} = f_{C\_Na} \cdot k_{C_2I_1} \cdot p(C)_{NaL} + k_{OI_1} \cdot p(O)_{NaL} - \left(k_{I_1C} + k_{I_1O} + k_{I_1I_2}\right) \cdot p(I_1)_{NaL} \quad Eq.S85$$

$$\frac{dp(I_2)_{NaL}}{dt} = f_{C\_Na} \cdot k_{C_2I_2} \cdot p(C)_{NaL} + k_{I_1I_2} \cdot p(I_1)_{NaL} + k_{I_sb} \cdot p(I_s)_{NaL} - \left(k_{I_2C} + k_{I_sf}\right) \cdot p(I_2)_{NaL} \qquad Eq.S86$$

$$\frac{dp(I_s)_{NaL}}{dt} = k_{I_sf} \cdot p(I_2)_{NaL} + k_{I_sf} \cdot p(C)_{NaL} - 2 \cdot k_{I_sb} \cdot p(I_s)_{NaL} \qquad Eq.S87$$

$$p(C)_{NaL} = 1.0 - p(I_s)_{NaL} - p(O)_{NaL} - p(I_1)_{NaL} - p(I_2)_{NaL} \qquad Eq.S88$$

$$k_{I_1I_2} = 0.00534 \qquad Eq.S89$$

$$k_{OI_1} = k_{OI_2} \qquad Eq.S90$$

$$k_{I_1O} = 0.01 \qquad Eq.S91$$

$$k_{I_1C} = k_{I_2C} \qquad Eq.S92$$

$$k_{C_2I_1} = k_{C_2I_2} \qquad Eq.S93$$

Inward rectifier potassium current (I_{K1})

The I_{K1} model developed by Yan and Ishihara (Yan and Ishihara, 2005) and Ishihara and Yan, (2007) was used in hVC model after modifying several parameters (Himeno et al., 2015).

$$I_{K1} = G_{K1} \cdot (V_m - E_K) \cdot p(O)_{K1} \qquad Eq.S94$$

$$G_{K1} = \frac{0.451773 \cdot \left(\frac{[K^+]_O}{5.4}\right)^{0.4}}{1 + exp\left(-\frac{[K^+]_O - 2.2}{0.6}\right)} \, nS/pF \qquad Eq.S95$$

$$p(O)_{K1} = pO_{mo} \quad + pO_{mode2} \qquad Eq.S96$$

Mode 1: the channel block by Mg^{2+} and spermine (SPM)

The SPM-block is a time-dependent process, while the Mg^{2+}-block is instantaneous.

$$Pbspm \underset{\beta \cdot pOMg}{\overset{\alpha}{\rightleftarrows}} 1 - Pbspm \qquad \text{Eq. S97}$$

$$\alpha_{Mg} = 12.0 \cdot exp\left(-\frac{V_m - E_K}{40}\right) \qquad Eq.S98$$

$$\beta_{Mg} = 28.0 \cdot exp\left(-\frac{V_m - E_K}{40}\right) \cdot [Mg^{2+}]_{cyt} \qquad Eq.S99$$

$$f_O = \frac{\alpha_{Mg}}{\alpha_{Mg} + \beta_{Mg}} \qquad Eq.S100$$

$$f_B = \frac{\beta_{mg}}{\alpha_{Mg} + \beta_{Mg}} \qquad Eq.S101$$

$$pO_{Mg} = f_O \cdot f_O \cdot f_O \qquad Eq.S102$$

$$pO_{Mg1} = 3.0 \cdot f_O \cdot f_O \cdot f_B \qquad Eq.S103$$

$$pO_{Mg2} = 3.0 \cdot f_O \cdot f_B \cdot f_B \qquad Eq.S104$$

$$pB_{Mg3} = f_B \cdot f_B \cdot f_B \qquad Eq.S105$$

$$\alpha_{SPM} = \frac{0.17 \cdot exp\left(-0.07 \cdot \left((V_m - E_K) + 8 \cdot [Mg^{2+}]_{cyt}\right)\right)}{1.0 + 0.01 \cdot exp\left(0.12 \cdot \left((V_m - E_K) + 8 \cdot [Mg^{2+}]_{cyt}\right)\right)} \qquad Eq.S106$$

$$\beta_{SP} = \frac{0.28 \cdot [SPM] \cdot exp\left(0.15 \cdot \left((V_m - E_K) + 8 \cdot [Mg^{2+}]_{cyt}\right)\right)}{1.0 + 0.01 \cdot exp\left(0.13 \cdot \left((V_m - E_K) + 8 \cdot [Mg^{2+}]_{cyt}\right)\right)} \qquad Eq.S107$$

$$\frac{dPb_{SPM}}{dt} = \beta_{SPM} \cdot pO_{Mg} \cdot (1 - Pb_{SPM}) - \alpha_{SPM} \cdot Pb_{SPM} \qquad Eq.S108$$

$$pO_{mode1} = 0.9 \cdot (1 - Pb_{SPM}) \cdot \left(pO_{Mg} + \frac{2}{3} pO_{Mg1} + \frac{1}{3} pO_{Mg}\right) \qquad Eq.S109$$

Mode 2: the channel block only by SPM

The channel is free from the Mg-block, and the SPM is instantaneous.

$$Pbspm \underset{Kd}{\longleftrightarrow} [SPM] \cdot (1 - Pbspm)$$

$$pO_{mode} = \frac{0.1}{1+\frac{[SPM]}{Kd}} \quad Kd = 40 \cdot exp\left(-\frac{V_m - E_K}{9.1}\right)\ mM \qquad Eq.S110$$

Delayed rectifier K+ current, fast component (I_{Kr})

We installed the I_{Kr} model developed by Ono and Ito (1995) (Ono and Ito, 1995), which well fitted the result of experimental I_{Kr} data of hiPSC-CMs (Ma et al., 2011)..

The current amplitude is described with an Ohmic equation.

$$I_{Kr} = G_{Kr} \cdot (V_m - E_K) \cdot p(O)_{Kr} \qquad Eq.S111$$

$$G_{Kr} = 0.049644 \cdot \left(\frac{[K^+]_o}{5.4}\right)^{0.2}\ nS/pF \qquad Eq.S112$$

The open probability of the channel is described with three gating parameters, y_1, y_2, and y_3, each of which is calculated by a two-state transition scheme.

$$p(O)_{Kr} = (0.6 \cdot y_1 + 0.4 \cdot y_2) \cdot y_3 \qquad Eq.S113$$

$$\frac{dy_N}{dt} = \alpha_{y_N} \cdot (1.0 - y_N) - \beta_{y_N} \cdot y_N, \qquad N = 1, 2, 3 \qquad Eq.S114$$

$$\alpha_{y_1} = \frac{1}{20 \cdot exp\left(-\frac{V_m + 6}{6}\right) + 5 \cdot exp\left(-\frac{V_m + 6}{150}\right)} \qquad Eq.S115$$

$$\beta_{y_1} = \frac{1}{160 \cdot exp\left(\frac{(V_m + 6)}{28}\right) + 200 \cdot exp\left(\frac{(V_m + 6)}{1000}\right)} + \frac{1}{2500 \cdot exp\left(\frac{(Vm + 6)}{20}\right)} \qquad Eq.S116$$

$$\alpha_{y_2} = \frac{1}{200 \cdot exp\left(-\frac{(V_m + 6)}{6.5}\right) + 20 \cdot exp\left(-\frac{(V_m + 6)}{150}\right)} \qquad Eq.S117$$

$$\beta_{y_2} = \frac{1}{1600 \cdot exp\left(\frac{(V_m + 6)}{28}\right) + 2000 \cdot exp\left(\frac{(V_m + 6)}{1000}\right)} + \frac{1}{10000 \cdot exp\left(\frac{(Vm + 6)}{20}\right)} \qquad Eq.S118$$

$$\alpha_{y_3} = \frac{1}{10 \cdot exp\left(\frac{(V_m + 6)}{17}\right) + 2.5 \cdot exp\left(\frac{(V_m + 6)}{300}\right)} \qquad Eq.S119$$

$$\beta_{y_3} = \frac{1}{0.35 \cdot exp\left(-\frac{(V_m + 6)}{17}\right) + exp\left(-\frac{(V_m + 6)}{75}\right)} \qquad Eq.S120$$

Delayed rectifier K$^+$ current, slow component (I_{Ks})

$$I_{Ks\_K} = P_{Ks} \cdot GHK_K \cdot p(O)_{Ks}\ ,\ P_{Ks} = 0.4\ (pA/pF/mM) \qquad Eq.S121$$

$$I_{Ks\_Na} = 0.04 \cdot P_{Ks} \cdot GHK_{Na} \cdot p(O)_{Ks} \qquad Eq.S122$$

$$p(O)_{Ks} = (O_v)^2 \cdot (0.99 \cdot O_C + 0.01) \qquad Eq.S123$$

The V$_m$-dependent gate

$$\alpha_{v_{Ks}} = \frac{1}{150 \cdot exp\left(-\frac{(V_m + 10)}{25}\right) + 900 \cdot exp\left(-\frac{(V_m + 10)}{200}\right)} \qquad Eq.S124$$

$$\beta_{v_{Ks}} = \frac{1}{1000 \cdot exp\left(\frac{(V_m + 10)}{13}\right) + 220 \cdot exp\left(\frac{(V_m + 10)}{50}\right)} \qquad Eq.S125$$

$$\frac{dO_v}{dt} = \alpha_{v_{Ks}} \cdot (1.0 - O_v) - \beta_{v_{Ks}} \cdot O_v \qquad Eq.S126$$

The Ca^{2+}-dependent gate

$$C1 \underset{\beta 3}{\overset{\alpha 3}{\rightleftarrows}} C2 \underset{\beta 2}{\overset{\alpha 2}{\rightleftarrows}} Oc$$

$$\frac{dO_c}{dt} = -\alpha_2 \cdot C_2 - \beta_2 \cdot O_c \qquad \text{Eq. S127}$$

$$\frac{dC_2}{dt} = \alpha_3 \cdot C_1 - \beta_3 \cdot C_2 - \alpha_2 \cdot C_2 + \beta_2 \cdot O_c \qquad \text{Eq. S128}$$

$$C_1 = 1.0 - C_2 - O_c \qquad \text{Eq. S129}$$

$$\alpha_3 = 0.0003, \qquad \beta_3 = 0.03 \qquad Eq.S130$$

$$\alpha_2 = 2.25 \cdot [Ca^{2+}], \qquad \beta_2 = 0.000296 \qquad Eq.S131$$

Transient outward K+ current (I_{Kto})

$$I_{Kto\_K} = P_{Kto} \cdot GHK_K \cdot p(O)_{Kto}\ , P_{Kto} = 0.01729\ (pA/pF/mM) \qquad Eq.S132$$

$$I_{Kto\_Na} = 0.09 \cdot P_{Kto} \cdot GHK_{Na} \cdot p(O)_{Kto} \qquad Eq.S40$$

$$p(O)_{Kto} = y_{1_{Kto}} \cdot y_{2_{Kto}} \qquad Eq.S41$$

$$\frac{dy_{1_{Kto}}}{dt} = \alpha_{y_1} \cdot \left(1.0 - y_{1_{Kto}}\right) - \beta_{y_1} \cdot y_{1_{Kto}}, \quad \frac{dy_{2_{Kto}}}{dt} = \alpha_{y_2} \cdot \left(1.0 - y_{2_{Kto}}\right) - \beta_{y_2} \cdot y_{2_{Kto}} \qquad Eq.S42$$

$$\alpha_{y_1} = \frac{1}{13 \cdot exp\left(-\frac{V_m}{22}\right)}, \qquad \beta_{y_1} = \frac{1}{2.1 \cdot exp\left(\frac{V_m}{90}\right)} \qquad Eq.S43$$

$$\alpha_{y_2} = \frac{0.5}{950 \cdot exp\left(\frac{V_m}{500}\right)}, \qquad \beta_{y_2} = \frac{0.5}{40 \cdot exp\left(-\frac{V_m}{9}\right) + 13 \cdot exp\left(-\frac{V_m}{1000}\right)} \qquad Eq.S44$$

Ultra-rapid K+ current (I_{Kur})

I_{Kur} model in a mouse ventricular cell model (Bondarenko et al., 2004) is used.

$$I_{Kur} = G_{Kur} \cdot (a_{ur})^3 \cdot i_{ur} \cdot (V_m - E_K) \qquad Eq.S45$$

$$G_{Kur} = 0.000113 \cdot \left(1 + \frac{1}{1 + exp\left(-\frac{V_m - 30}{59}\right)}\right) nS/pF \qquad Eq.S46$$

$$\frac{da_{ur}}{dt} = \alpha_{ur} \cdot (1.0 - a_{ur}) - \beta_{ua} \cdot a_{ur}, \qquad \frac{di_{ur}}{dt} = \alpha_{ui} \cdot (1.0 - i_{ur}) - \beta_{ui} \cdot i_{ur} \qquad Eq.S47$$

$$\alpha_{ua} = \frac{1}{0.65 \cdot exp\left(-\frac{(V_m + 19)}{8.5}\right) + exp\left(-\frac{(V_m - 21)}{59}\right)}, \qquad \beta_{ua} = \frac{1}{0.65 \cdot \left(2.5 + exp\left(\frac{(V_m + 91)}{17}\right)\right)} \quad Eq.S48$$

$$\alpha_{ui} = \frac{1}{21 + exp\left(-\frac{(V_m - 185)}{28}\right)}, \qquad \beta_{ui} = exp\left(\frac{V_m - 158}{16}\right) \qquad Eq.S49$$

Time-independent currents

All these currents are from Takeuchi *et al.* (Takeuchi et al., 2006) as described in Asakura *et al.* (Asakura et al., 2014).

Background Ca2+ current (I_{bCa})

$$I_{bCa\_a} = P_{bCa\_a} \cdot 2 \cdot GHK_{Ca}, \qquad a = (blk, iz) \qquad Eq.S50$$

$$P_{bCa} = 0.00125 \ \ (pA/pF/mM) \qquad Eq.S51$$

Background non-selective cation current (I_{bNSC})

$$I_{bNSC\_X} = P_{bNSC_X} \cdot GHK_X, \qquad X = (K, Na) \qquad Eq.S52$$

$$P_{bNSC\_Na} = 0.000182875, P_{bNSC\_K} = 0.4 \cdot P_{bNSC\_Na}(pA/pF/mM) \qquad Eq.S53$$

$$I_{bNSC} = I_{bNSC\_K} + I_{bNSC\_Na} \qquad Eq.S54$$

Calcium-activated background cation current ($I_{l(Ca)}$)

$$p(O)_a = \frac{1.0}{1.0 + \left(\frac{0.0012}{[Ca^{2+}]_a}\right)^3} \qquad Eq.S148$$

$$I_{l(Ca)\_X\_a} = P_{l(Ca)\_X\_a} \cdot f_{l(Ca)\_X\_a} \cdot GHK_X \cdot p(O)_a, \qquad X = (Na, K), \qquad a = (blk, iz) \quad Eq.S149$$

$$P_{l(Ca)\_Na} = 0.01375 \ (pA/pF/mM) \qquad Eq.S150$$

$$P_{l(Ca)\_K} = P_{l(Ca)\_Na} \ (pA/pF/mM) \qquad Eq.S151$$

Fraction of $I_{l(Ca)}$

$$f_{l(Ca)\_iz} = 0.1, f_{l(Ca)\_blk} = 0.9 \qquad Eq.S152$$

$$I_{l(Ca)} = I_{l(Ca)\_Na\_iz} + I_{l(Ca)\_K\_iz} + I_{l(Ca)\_Na\_blk} + I_{l(Ca)\_K\_blk} \qquad Eq.S153$$

ATP-sensitive potassium current (I_{KATP})

$$p(O)_{KATP} = \frac{0.8}{1.0 + \left(\frac{[ATP]_{cyt}}{0.1}\right)^2} \qquad Eq.S154$$

$$\chi_{KATP} = 0.0236 \cdot ([K^+]_o)^{0.24} \qquad Eq.S155$$

$$I_{KATP} = G_{KATP} \cdot (V_m - E_K) \cdot p(O)_{KATP} \cdot \chi_{KATP} \qquad Eq.S156$$

$$G_{KATP} = 18.75 \qquad Eq.S157$$

Na^+/K^+ pump current (I_{NaK})

The Na^+/K^+ pump model developed by Oka *et al.* (Oka et al., 2010) on the framework of Smith and Crampin (Smith and Crampin, 2004) was used after adjusting the amplitude as indicated in the manuscript in Eq. 13.

Na^+/Ca^{2+} exchange current (I_{NCX})

The NCX model developed by Takeuchi *et al.* (Takeuchi et al., 2006) was used after adjusting the amplitude as indicated in the manuscript in Eq. 14.

CaRU

The model of CaRU described in the hVC model was used. The model structure of CaRU is shown in Fig. S1. Derivation of the instantaneous $[Ca^{2+}]_{nd}$, which is sensed by both LCC and RyRs for inactivation and activation, respectively, was originally given in Hinch 2004 and modified by Himeno et al. 2015. In short, the volume of *nd* was assumed to be virtually zero so that the $[Ca^{2+}]_{nd}$ can be given by the instantaneous equation Eq. S158,

$$[Ca]_{nd} = \frac{[Ca]_{jnc} + \frac{g_R}{g_d}\cdot[Ca]_{SRrl} + \frac{g_L}{g_d}\cdot\frac{\delta V\cdot e^{-\delta V}}{1-e^{-\delta V}}\cdot[Ca]_o}{(1+\frac{g_R}{g_d}+\frac{g_L}{g_d}\cdot\frac{\delta V\cdot e^{-\delta V}}{1-e^{-\delta V}})} \qquad \text{Eq. S158}$$

where Ca^{2+} fluxes through LCC and RyR, and diffusion from *nd* to *jnc* (J_L, J_R and J_D) were given as Eqs. S134-136.

$$J_L = g_L\cdot\frac{\delta V\cdot e^{-\delta V}}{1-e^{-\delta V}}\cdot([Ca]_o-[Ca]_{nd}) \qquad \text{Eq. S159}$$

$$J_R = g_R\cdot([Ca]_{SRrl}-[Ca]_{nd}) \qquad \text{Eq. S160}$$

$$J_D = g_D\cdot([Ca]_{nd}-[Ca]_{jnc})$$

Eq. S161

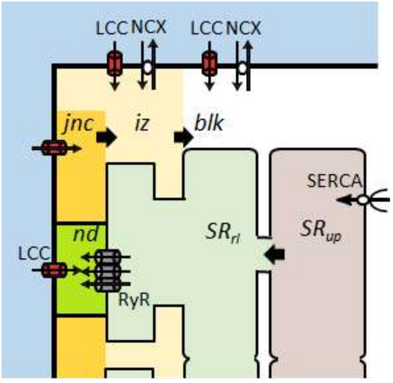

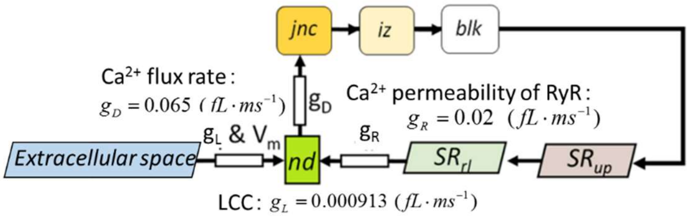

Fig. S1 Model structure of CaRU in relation to three Ca^{2+} diffusion compartments (left) and direction of Ca^{2+}diffusion (right) within the cell model. The L-type Ca^{2+} channel (LCC) and NCX are located on the sarcolemma, SERCA and RyRs are on the SR membrane. A single CaRU consists of a hypothetical LCC and a couplon (a cluster of RyRs) in the junctional cleft (filled with lime green), and individual CaRUs are spatially separated from its neighbors by jnc. The inset on the right shows schematic presentation of the diffusion pathway of Ca^{2+} from the Ca^{2+} sources (SR or extracellular space) to the sink (nd) and then to the cytoplasm (jnc, iz and blk). J_L, J_R and g_D represent permeability of single LCC and RyR, and Ca^{2+} flux rate from nd to jnc, respectively.

Sarcoplasmic reticulum Ca^{2+} pump (SERCA) current (J_{SERCA})

The three-state model developed by Tran *et al.* (Tran et al., 2009) was used after several minor modifications as described in Asakura *et al.* (Asakura et al., 2014). The limiting amplitude of J_{SERCA}, Amp_{SERCA}, was modified.

Rate of change in the membrane potential and ion concentrations

Membrane potential

$$\frac{dV_m}{dt} = -(I_{tot\_cell} + I_{app}) \qquad Eq.S162$$

$$I_{tot\_cell} = I_{tot\_Ca} + I_{tot\_Na} + I_{tot\_K} \qquad Eq.S163$$

$$I_{tot\_Ca} = I_{tot\_Ca\_jnc} + I_{tot\_Ca\_iz} + I_{tot\_Ca\_blk} \qquad Eq.S164$$

$$I_{tot\_Ca\_jnc} = I_{CaL\_Ca\_LR} + I_{CaL\_Ca\_L0} \qquad Eq.S165$$

$$I_{tot\_Ca\_iz} = I_{CaL\_Ca\_iz} + I_{NCX\_Ca\_iz} + I_{Cab\_iz} \qquad Eq.S166$$

$$I_{tot\_Ca\_blk} = I_{CaL\_Ca\_blk} + I_{CaT} + I_{Cab\_blk} + I_{NCX\_Ca\_blk} \qquad Eq.S167$$

$$I_{tot\_Na} = (I_{CaL\_Na\_jnc} + I_{CaL\_Na\_iz} + I_{CaL\_Na\_blk}) + (I_{NCX\_Na\_iz} + I_{NCX\_Na\_blk}) + (I_{Ks\_Na\_iz} + I_{Ks\_Na\_blk}) + I_{NaT\_Na} + I_{NaL\_Na} + I_{NaK\_Na} + I_{Kto\_Na} + I_{bNSC\_Na} + (I_{LCCa\_Na\_iz} + I_{LCCa\_Na\_blk}) + I_{st\_Na} + I_{ha\_Na} \quad Eq.S168$$

$$I_{tot\_K} = (I_{CaL\_K\_jnc} + I_{CaL\_K\_iz} + I_{CaL\_K\_blk}) + I_{NaT\_K} + I_{NaL\_K} + I_{K1} + I_{Kur} + I_{Kpl} + I_{Kr} + (I_{Ks\_K\_iz} + I_{Ks\_K\_blk}) + I_{Kto\_K} + I_{KATP\_K\_cyt} + I_{bNSC\_K} + (I_{LCCa\_K\_iz} + I_{LCCa\_K\_blk}) + I_{NaK\_K} + I_{KACh} + I_{st\_K} + I_{ha\_K} \quad Eq.S169$$

Ion concentrations

$$\frac{d[Ca^{2+}_{total}]_{jnc}}{dt} = -\frac{I_{tot\_Ca\_jnc} \cdot C_m}{V_{jnc} \cdot 2 \cdot F} + \frac{J_{Ca\_rel}}{V_{jnc}} - \frac{J_{Ca\_jnciz}}{V_{jnc}} \quad Eq.S170$$

$$\frac{d[Ca^{2+}_{total}]_{iz}}{dt} = -\frac{I_{tot\_Ca\_iz} \cdot C_m}{V_{iz} \cdot 2 \cdot F} + \frac{J_{Ca\_jnciz}}{V_{iz}} - \frac{J_{Ca\_izblk}}{V_{iz}} \quad Eq.S171$$

$$\frac{d[Ca^{2+}_{total}]_{blk}}{dt} = -\frac{I_{tot\_Ca\_blk} \cdot C_m}{V_{blk} \cdot 2 \cdot F} + \frac{J_{Ca\_izblk}}{V_{blk}} - \frac{J_{Ca\_SERCA}}{V_{blk}} \quad Eq.S172$$

$$\frac{d[Ca^{2+}]_{SRup}}{dt} = \frac{J_{Ca\_SERCA}}{V_{SRup}} - \frac{J_{trans\_SR}}{V_{SRup}} \quad Eq.S173$$

$$\frac{d[Ca^{2+}_{total}]_{SRrl}}{dt} = \frac{J_{trans\_SR}}{V_{SRrl}} - \frac{J_{rel\_SR}}{V_{SRrl}} \quad Eq.S174$$

$$\frac{d[Na^{+}]_{i}}{dt} = -\frac{I_{tot\_Na} \cdot C_m}{V_{cyt} \cdot F} \quad Eq.S175$$

$$\frac{d[K^{+}]_{i}}{dt} = -\frac{(I_{tot\_K} + I_{app}) \cdot C_m}{V_{cyt} \cdot F} \quad Eq.S176$$

Contraction

The original model of Negroni and Lascano (Negroni and Lascano, 2008) was used. The magnitude of F_b is given in a unit of mN·mm^{-2}. The binding of Ca^{2+} to a troponin system (TS) having 3 Ca^{2+} binding sites (given in μM) was included in the equation of determining the concentration of free Ca^{2+} in the bulk compartment.

$$[Ca^{2+}]_{blk} = [Ca^{2+}_{total}]_{blk} - \left([CaMCa] + [TnChCa] + [SRCa] + \frac{3 \cdot ([TSCa_3] + [TSCa_3^{\sim}] + [TSCa_3^{*}])}{1000}\right)$$

$$Eq.S177$$

Fitting the hiPSC_CM model to ion channel data of hiPSC-CMs

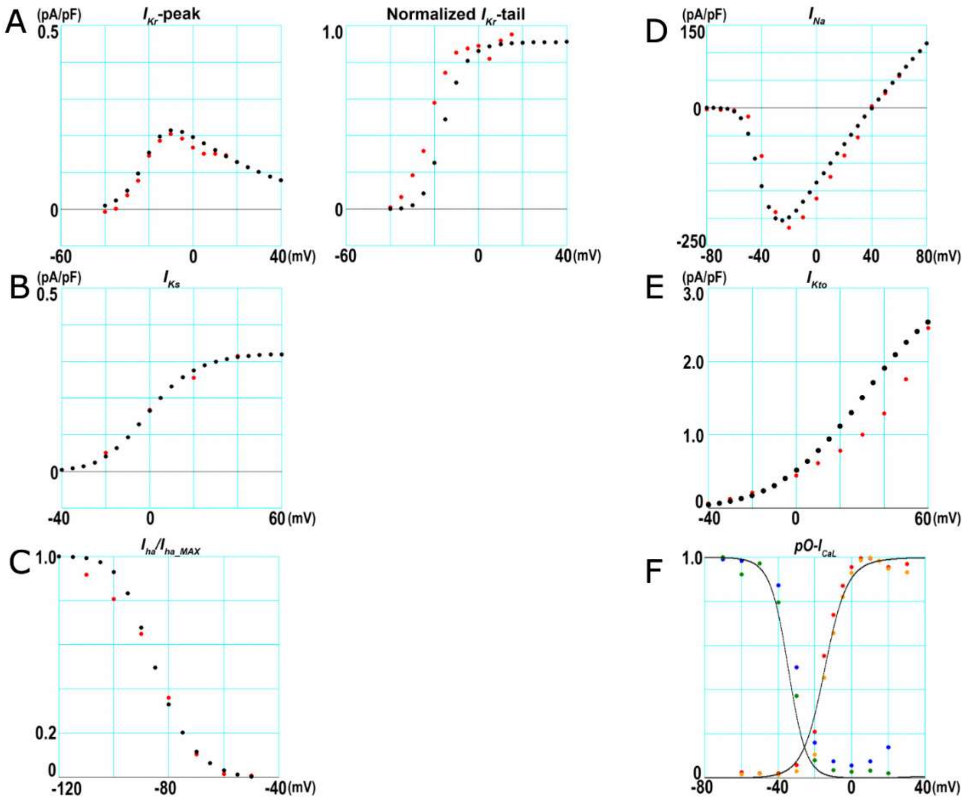

Fig. S2 Comparison of previously reported experimental results with mathematical models fitted to them. hiPSC-CMs mathematical model was constructed on the basis of hVC model and modified for experimental data from hiPSC-CMs. A, B, C, D, and E: Red points show experimental data from Ma et al., 2011, and black points exhibit fitting results of mathematical modeling. F: Red and blue points show experimental data from Ma et al., 2011. Orange and green points in F are our original experimental results. Black traces exhibit fitting results of mathematical modeling.

Pattern Search Algorithm

Pseudo code of the pattern search algorithm used in the research is shown in Fig. S3.

```
PatternSearch():
  Stp = 1/100;
  RedFct = 1/4;
  EPS = 1.0E+37;
  Eval = 0;
  for (i = 0; i < NumPara; i++) {
    NP[i] = BP[i] = RandomBP();
  }

  MIN = MSE = givenEquation(NP);
  Eval++;

  do {
    Fails = EXPLORER();
    while (Fails != NumPara) {
      for (i = 0; i < NumPara; i++) {
        Advance[i] = NP[i] - BP[i];
      }
      do {
        for (i = 0; i < NumPara; i++) {
              BP[i] = NP[i];
          NP[i] = BP[i] + Advance[i];
          if (Advance[i] * Stp[i] <= 0) {
            Stp[i] = -Stp[i];
          }
        }
        If Eval > EvalMax {
          return;
        }
        MinSto = MIN;
        MIN = MSE = givenEquation(NP);
        Fails = EXPLORE();
        if (MIN <= MinSto) {
          for (i = 1; i < NumPara; i++) {
            Advance[i] = NP[i] - BP[i];
          }
          maxAdvance = max(Advance[]);
        }
      } while ((MIN <= MinSto) && (maxAdvance > EPS));
      MIN = MinSto;
      for (i = 0; i < NumPara; i++) {
        NP[i] = BP[i];
      }
      Fails = EXPLORE();
    }
    Stp = Stp * RedFct;
  } while (Stp / RedFct > CrtStp);
}
```

```
EXPLORE() {
  Fails = 0;
  for (i = 0; i < NumPara; i++) {
    HOME = NP[i];
    NP[i] = HOME + Stp;
    MSEp = givenEquation(NP);
    NP[i] = HOME - Stp;
    MSEn = givenEquation(NP);
    minMSE = min(MSEp, MSEn);
    if (minMSE < MSE) {
      if (MSEp < MSEn) {
        NP[i] = HOME + Stp;
      } else {
        NP[i] = HME - Stp;
      }
      MSE = minMSE;
    } else {
      NP[i] = HOME;
      Fails++;
    }
  }
  return Fails;
}
```

Fig. S3 Pseudo code of the pattern search algorithm explained in Sec. 3.3. Function EXPLORER() searches for the set of sf_x *that gives smaller MSE by evaluating* $sf_x \pm stp$ *for each* sf_x*s. Function givenEquation() calculates MSE with* sf_x *given by the variable NP[].*

Results in less negative MDP cell (Cell 38)

Mapping the magnitude of MSE over the 9 global parameter space

Global random test of nine parameters for a cell with MDP less than -75 mV (Cell 86) was shown in the main manuscript. Here results of same random test for a cell with MDP higher than -75 mV (Cell 38) is shown in Fig S4. Similar with Cell 86, single peak was observed for all the selected currents, and no other local solution was found in this global range.

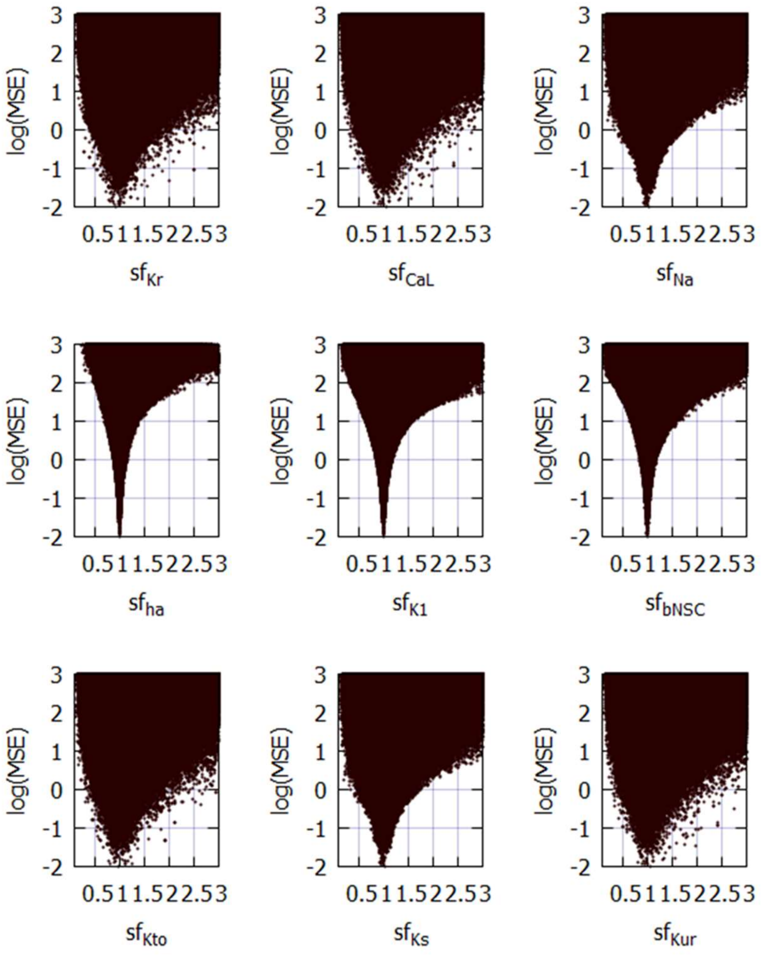

Fig. S4. Nine-parameter random test of cell-38 for the MSE in the sf_x-space. The relative value of each current component is plotted on the x-axis, and the log(MSE) is plotted on the y-axis. 37,070,869 points were plotted. The random range for setting the initial value of sf_x were put to the target value of 0.1-10.

The four-parameter orp test in cell 38

As shown in the nine parameter orp test, significant currents are I_{Kr}, I_{CaL}, I_{Kur} and I_{bNSC} in Cell 38, which can be selected from the physiological consideration. Results of this four parameter orp test is shown in Fig S5. We can find clear convergence of selected four currents.

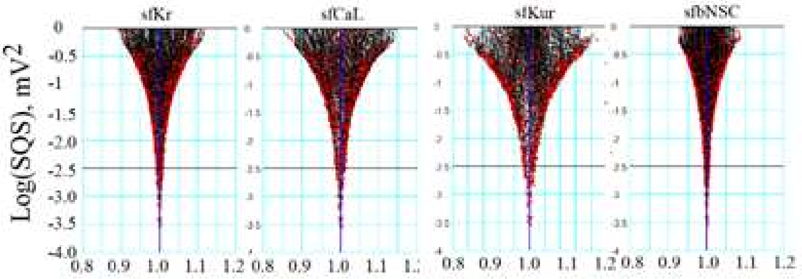

Fig. S5. Convergence of sf_x in the orp test for Cell38. The ordinate is the Log(MSE) and the abscissa the normalized amplitude of sf_x; x stands for I_{Kr}, I_{CaL}, I_{Kur} and I_{bNSC}. Points indicate sf_x obtained in 838 runs of PS optimization.

New findings of the I_{Kr} blockade by the E4031

The application of the PO method to the selective I_{Kr} blocker E4031, preliminary findings.

So far, the cell-specific models were created by Lei et al. (2017). They measured I_{Na}, I_{CaL} and the whole cell outward current by applying the voltage clamp technique to 22 iPSC-CMs (cell line Cor.4U). They also optically measured APDs in other iPSC-CMs under both control and drug-applications. To create cell-specific models, they tailored the original model of Paci et al. (2013) simply by optimizing the maximum conductances of ion channels obtained by the voltage clamp. The method used in their optimization was CMA-ES, which is a mixture of stochastic and gradient based optimization methods. They found that tailored cell-specific models obtained by the relatively simple approach predicted the changes in APDs induced by drug applications better than the original model. They suggested the need to use more intricate methods to characterize differences in outward currents.

We applied a saturating concentration of E4031 (1 μM) to hiPSC-CMs, which were purchased from Myoridge Co. Ltd., Kyoto, Japn (CarmyA, H-011106). These cells were derived from the same hiPSC line (253G1) but differentiated into cardiomyocytes using a protein-free chemically defined medium, which is different from the differentiation procedure using a protein-based cytokines and growth factors in Department of Cardiovascular Medicine, Kyoto University. The time course of the response to the I_{Kr}-block was recorded in four hiPSC-CMs (TC06, TC11, TC12 and TC13) as demonstrated in Fig. S7A. The cell-specific model was determined first by applying the new PO method to a representative AP configuration in the control obtained before the application of I_{Kr} blockade in each cell.

Fig. S6 shows variable AP configurations of the cell-specific model (black trace) superimposed on corresponding experimental AP records obtained in the four hiPSC-CMs. See

Table S5 for the AP metrics as well as the MSE between the target AP and the model output at the end of the parameter optimization. When compared among the four cells, the least negative MDP and the highest rate of SDD in TC06 are attributed mainly to the larger I_{bNSC} than the other extreme of negative MDP in TC13 (Fig. S6C). On the other hand, the largest AP amplitude and modest SDD in TC13 are explained by the low current density (i.e. low membrane conductance) during SDD (Fig. S6B & C). The highest rate of the phase 2 repolarization in TC11 is attributed to the largest I_{Kto}. The amplitude of I_{ha} is large in TC06, but is much less visible in other cells.

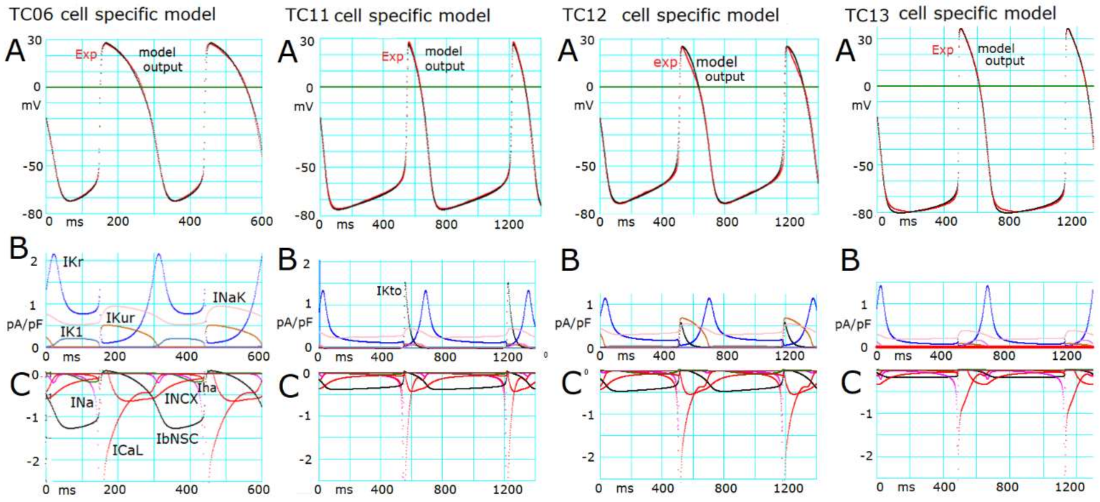

Fig. S6 Comparison of ionic currents underlying the variable configurations of AP in four hiPSC-CMs. Fig. S6A shows the AP configurations. The experimental record (red trace) was superimposed by the model-output (black) of each cell-specific model. The Figs S6B and S6C show the outward and inward currents of the cell specific model, respectively. Each current is depicted in different colors as indicated in Fig. S6B & C of TC06 and in TC11 (I_{Kto}). Note, the time scale for TC06 is shorter than others to demonstrate sequential two APs.

Table S5 AP metrics of the cell specific models

CL; the cycle length of the spontaneous AP. MDP; the maximum diastolic potential. APD; the duration of the AP measured at -20 mV.

	CL (ms)	OS (mV)	MDP (ms)	APD(ms) at -20mV	log(MSE) (mV^2)
TC06	290	27.3	-72.1	141	-0.139
TC11	663	27.6	-75.5	116	-0.135
TC12	675	24.2	-74.2	163	-0.229
TC13	641	36	-79.5	153	0.359

To reconstruct the experimental time-course of E4031 influence on the spontaneous AP, the I_{Kr} amplitude of the cell-specific model was gradually decreased using the exponential function (Eq. S178 described in Legend of Fig. S7). The computer model output well simulated the experimental time course of changes in OS and MDP (Fig. S7A, B). The experimental recording, however, showed obvious fluctuations in contrast to the smooth time course OS and MDP modification in the model output. Although we did not examine the mechanisms of these fluctuations, the rate of phase 3 rapid repolarization (Fig. S7C) was progressively decreased by the reduction of the I_{Kr} amplitude as shown in Fig. S7D. Close inspection of the extent of the positive shift of MDP revealed marked difference between TC06 (beyond the arbitrary reference of horizontal red line, -50 mV in Fig. S7A & B) and TC13 (below -50 mV) in both experimental record and the model output. While TC11 and TC12 showed moderate MDP depolarization to

about -50 mV. On the other hand, no obvious difference was observed among the four cells in the relative extent of decrease in the I_{Kr} amplitude if compared at four different recording points shown by the colored circles in panels B. The different responses to I_{Kr}-blockade among the four cells despite similar extent of decrease in the I_{Kr} amplitude were most probably attributed to the variation of the current compositions among hiPSC-CMs as shown in Figs. S6B & C.

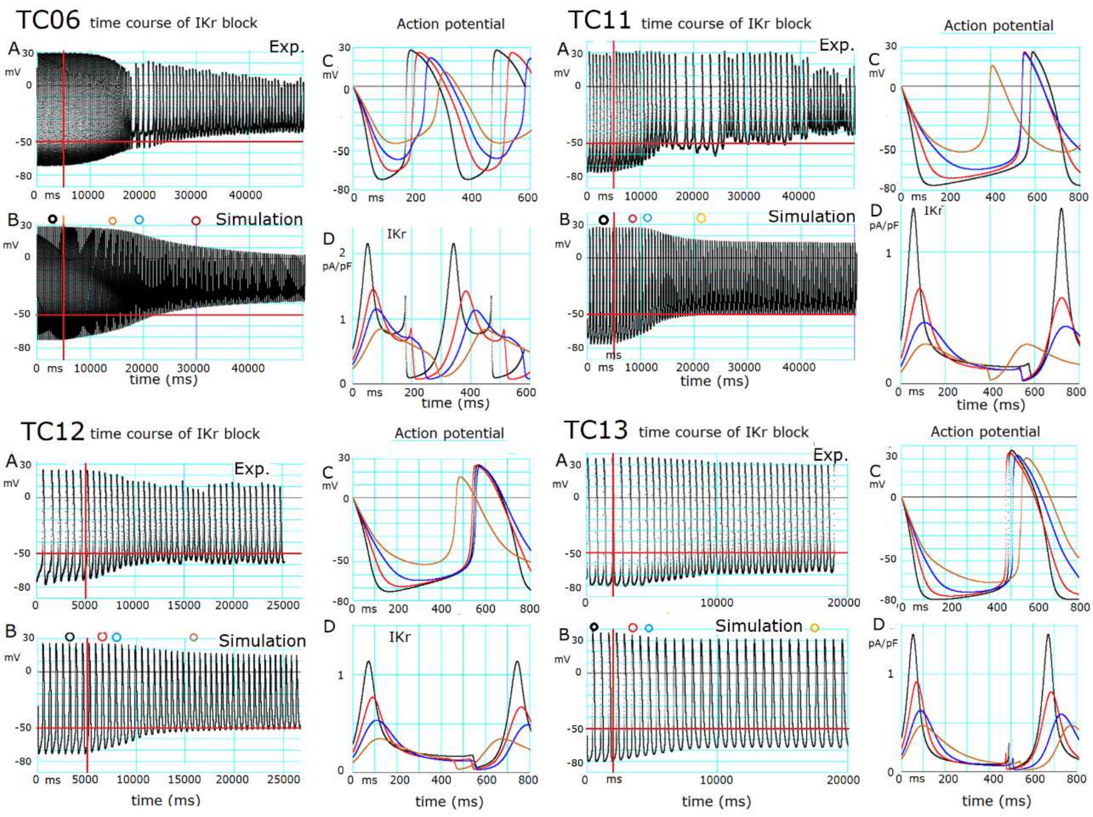

Fig. S7 Reconstruction of the hiPSC-CM response to the I_{Kr} blockade using the cell-specific model.

A; the experimental (chart) recording of V_m, in which the test solution of I_{Kr}-selective blocker E4031 was assumed to reach the cell in the recording chamber at around 5000 msec (red vertical line). The MDP of AP gradually depolarized reflecting the progressive blockade of I_{Kr}. B; The response of cell-specific model is demonstrated in panel C on an expanded timescale. The V_m (C) and I_{Kr} (D) of the cell-specific model are depicted with the corresponding colors of APs, which are selected to represent the progressive I_{Kr}-blockade as indicated by open circles in panel B.

Model simulation of the I_{Kr}-blockade: In the simulation (B), the I_{Kr} block started at 5 s by using exponential time constants of 12, 4, 2.65 and 1.4 s and infinite sf_xs of 0.35, 0.31, 0.35 and 0.368 in Eq. S178, for TC06, TC11, TC12 and TC13 cell-specific models, respectively. In experiments, a saturating dose of E4031 (1 μM) was applied by switching the perfusate of the recording chamber (Fig. S7A). The reconstruction of the drug-response of the cell-specific model is shown in Fig. S7B. Since the effective concentration of E4031 in the vicinity of the target cell was not available in the experimental recording chamber, it was necessary to adjust the time course of the model output by using a conventional exponential function.

$$sf_{Kr}(t+dt) = sf_{Kr}(\infty) - (sf_{Kr}(\infty) - sf_{Kr}(t)) \times e^{-dt/\tau}, \qquad \text{(Eq. S178)}$$

where $sf_{Kr}(t)$: a time-varying scaling factor I_{Kr} at a recording time t, $sf_{Kr}(\infty)$: at an infinite time, $sf_{Kr}(t+dt)$:a partial blockade of I_{Kr} at time t + dt, dt: a time step of numerical integration, and τ: a time constant.

These findings are roughly consistent with those predicted by the analyses using the model obtained by the conventional manual fitting of the parameter to the average of experimental data. To note, however, the use of the cell-specific model obtained by the PO method enabled the analysis in individual hiPSC-CM specimens, which usually showed a large variety in AP configurations as well as AP cycles. In the analyses of using an average model, it may be difficult to predict the individual cell response to the drug application in agreement with Lei et al (2017).

Ideally, the application of PO method to experimental I_{Kr} blockade data should be able to provide sf_{Kr} magnitude decreased by a I_{Kr}-selective blocker leaving other sf_{x}s remained intact. In our preliminary application of the PO method to the time course of I_{Kr} blockade, however, we failed to get the selective blockade except for a few successful trials (not shown). It might be suggested that the central assumption, that the kinetics of the ion channels remained intact, did not hold true in applying the PO method when any secondary influences of the I_{Kr} blockade were induced. Obviously, we need to make further tuning of the PO method or modifying the experimental protocol in the future studies.

Nomenclature of the ionic currents

*Table S5 Molecular determinants for each current and their expression in iPS-CMs hiPS, human iPS cell line (253G1); hiPS-CM, human iPS cell-derived cardiomyocytes; FH, fetal human heart tissues; AH, adult human heart tissues. RNA expression profiles in hiPS, hiPS-CM, FH and AH were obtained from the Gene Expression Omnibus (GEO) public database (accession: GSE154580 GEO Accession viewer (nih.gov)). * This is presumed by recent studies (Toyoda et al., 2017; Toyoda et al., 2018).*

Current		Protein	Gene	RNA expression (#GSE154580) hiPS	hiPS-CM	FH	AH
I_{Kr}	rapid component of delayed rectifier K^+ current	$K_v11.1$	*KCNH2*	29.50	244.41	60.75	116.85
I_{K1}	inward rectifier K^+ current	$K_{ir}2.1$	*KCNJ2*	1.18	6.16	69.03	48.00
I_{CaL}	L-type Ca^{2+} current	$Ca_v1.2$<br>$Ca_v1.3$	*CACNA1C*<br>*CACNA1D*	0.00<br>0.34	6.58<br>3.37	3.02<br>1.39	2.84<br>0.23
I_{bNSC}	background nonselective cation current	unidentified	unidentified				
I_{ha}	hyperpolarization activated currenIt	HCN4<br>HCN1<br>HCN2	*HCN4*<br>*HCN1*<br>*HCN2*	54.48<br>7.19<br>2.27	491.55<br>17.02<br>11.16	19.97<br>0.85<br>0.16	28.13<br>1.32<br>10.61
I_{Ks}	slow component of delayed rectifier K^+ current	Kv7.1<br>(+KCNE1)	*KCNQ1*<br>(+*KCNE1*)	4.16<br>(0.00)	86.48<br>(0.19)	60.75<br>(0.93)	53.6<br>(4.55)
I_{Kto}	transient outward K^+ current	$K_v4.3$<br>$K_v1.4$	*KCND3*<br>*KCNA4*	0.00<br>0.08	0.00<br>8.45	0.23<br>4.80	0.28<br>10.97
I_{Kur}	ultra-rapid component of rectifier K^+ current	$K_v1.5$	*KCNA5*	0.33	0.56	3.25	6.60
I_{Na}	sum of voltage-gated Na^+ current in transient and late modes (I_{NaT} + I_{NaL})	$Na_v1.5$	*SCN5A*	15.00	388.97	58.42	274.9
I_{CaT}	T-type Ca^{2+} current	$Ca_v3.2$<br>$Ca_v3.1$	*CACNA1H*<br>*CACNA1G*	26.98<br>0.00	1.77<br>0.64	113.76<br>0.00	4.30<br>0.00
I_{st}	sustained inward current	Cav1.3*	*CACNA1D*	0.34	3.37	1.39	0.23
I_{NaK}	Na^+/K^+ pump current	Na,K-ATPase<br>α subunit<br>β subunit	*ATP1A1*<br>*ATP1B1*	603.19<br>80.03	1522.44<br>601.05	631.26<br>480.27	562.90<br>539.60
I_{NCX}	Na^+/Ca^{2+} exchanger current	NCX1	*SLC8A1*	0.14	1509.25	266.76	560.11

References

Asakura, K., Cha, C. Y., Yamaoka, H., Horikawa, Y., Memida, H., Powell, T., et al. (2014). EAD and DAD mechanisms analyzed by developing a new human ventricular cell model. *Progress in Biophysics and Molecular Biology* 116, 11–24. doi:10.1016/j.pbiomolbio.2014.08.008.

Bondarenko, V. E., Szigeti, G. P., Bett, G. C. L., Kim, S.-J., and Rasmusson, R. L. (2004). Computer model of action potential of mouse ventricular myocytes. *American Journal of Physiology - Heart and Circulatory Physiology* 287, H1378–403. doi:10.1152/ajpheart.00185.2003.

Guo J, Ono K, Noma A. (1995) A sustained inward current activated at the diastolic potential range in rabbit sino-atrial node cells. *J. Physiol. (Lond.)* 483:1-13. doi: 10.1113/jphysiol.1995.sp020563

Ferreira, G., Yi, J., Ríos, E., and Shirokov, R. (1997). Ion-dependent inactivation of barium current through L-type calcium channels. *The Journal of General Physiology* 109, 449–461. doi:10.1085/jgp.109.4.449.

Grandi, E., Pasqualini, F. S., and Bers, D. M. (2010). A novel computational model of the human ventricular action potential and Ca transient. *J. Mol. Cell. Cardiol.* 48, 112–121. doi:10.1016/j.yjmcc.2009.09.019.

Himeno, Y., Asakura, K., Cha, C. Y., Memida, H., Powell, T., Amano, A., et al. (2015). A Human Ventricular Myocyte Model with a Refined Representation of Excitation-Contraction Coupling. *Biophysical Journal* 109, 415–427. doi:10.1016/j.bpj.2015.06.017.

Hinch R. (2004). A mathematical analysis of the generation and termination of calcium sparks. *Biophys J* 86(3), 1293-307. doi: 10.1016/S0006-3495(04)74203-4.

Ishihara K, Yan DH. (2007). Low-affinity spermine block mediating outward currents through Kir2.1 and Kir2.2 inward rectifier potassium channels. *J Physiol* 583(Pt 3), 891-908. doi: 10.1113/jphysiol.2007.136028.

Ma, J., Guo, L., Fiene, S. J., Anson, B. D., Thomson, J. A., Kamp, T. J., et al. (2011). High purity human-induced pluripotent stem cell-derived cardiomyocytes: electrophysiological properties of action potentials and ionic currents. *Am. J. Physiol. Heart Circ. Physiol.* 301, H2006–17. doi:10.1152/ajpheart.00694.2011.

Maruoka F, Nakashima Y, Takano M, Ono K, Noma A. (1994). Cation-dependent gating of the hyperpolarization-activated cation current in the rabbit sino-atrial node cells. *J Physiol* (Lond) 477, 423–435. doi:10.1113/jphysiol.1994.sp020204

Mitsuiye T, Guo J, Noma A. (1999) Nicardipine-sensitive Na^+-mediated single channel currents in guinea-pig sinoatrial node pacemaker cells. *JPhysiol* (Lond) 521: 69-79. doi:10.1111/j.1469-7793.1999.00069.x

Negroni, J. A., and Lascano, E. C. (2008). Simulation of steady state and transient cardiac muscle response experiments with a Huxley-based contraction model. *J. Mol. Cell. Cardiol.* 45, 300–312. doi:10.1016/j.yjmcc.2008.04.012.

Noma A, Irisawa H. (1976). Membrane currents in the rabbit sinoatrial node cell as studied by the double microelectrode method. *Pflugers Arch.* 364(1):45–52. doi:10.1007/bf01062910.1976.

Oka, C., Cha, C. Y., and Noma, A. (2010). Characterization of the cardiac Na+/K+ pump by development of a comprehensive and mechanistic model. *Journal of Theoretical Biology* 265, 68–77. doi:10.1016/j.jtbi.2010.04.028.

Ono, K., and Ito, H. (1995). Role of rapidly activating delayed rectifier K+ current in sinoatrial node

pacemaker activity. *Am. J. Physiol.* 269, H453–62. doi:10.1152/ajpheart.1995.269.2.H453.

Shirokov, R., Levis, R., Shirokova, N., and Ríos, E. (1993). Ca(2+)-dependent inactivation of cardiac L-type Ca2+ channels does not affect their voltage sensor. *The Journal of General Physiology* 102, 1005–1030. doi:10.1085/jgp.102.6.1005.

Smith, N. P., and Crampin, E. J. (2004). Development of models of active ion transport for whole-cell modelling: cardiac sodium-potassium pump as a case study. *Progress in Biophysics and Molecular Biology* 85, 387–405. doi:10.1016/j.pbiomolbio.2004.01.010.

Takeuchi, A., Tatsumi, S., Sarai, N., Terashima, K., Matsuoka, S., and Noma, A. (2006). Ionic mechanisms of cardiac cell swelling induced by blocking Na^+/K^+ pump as revealed by experiments and simulation. *The Journal of General Physiology* 128, 495–507. doi:10.1085/jgp.200609646.

Toyoda F, Mesirca P, Dubel S, Ding WG, Striessnig J, Mangoni ME, Matsuura H. (2017) Cav1.3 L-type Ca^{2+} channel contributes to the heartbeat by generating a dihydropyridine-sensitive persistent Na^+ current. Sci. Rep. Aug 11;7(1):7869. doi: 10.1038/s41598-017-08191-8.

Toyoda F, Ding WG, Matsuura H. (2018). Heterogeneous functional expression of the sustained inward Na + current in guinea pig sinoatrial node cells. *Pflugers Arch* 470(3), 481-490. doi: 10.1007/s00424-017-2091-y.

Tran, K., Smith, N. P., Loiselle, D. S., and Crampin, E. J. (2009). A thermodynamic model of the cardiac sarcoplasmic/endoplasmic Ca(2+) (SERCA) pump. *Biophysical Journal* 96, 2029–2042. doi:10.1016/j.bpj.2008.11.045.

Yan, D.-H., and Ishihara, K. (2005). Two Kir2.1 channel populations with different sensitivities to Mg(2+) and polyamine block: a model for the cardiac strong inward rectifier K(+) channel. *J. Physiol. (Lond.)* 563, 725–744. doi:10.1113/jphysiol.2004.079186.

Yanagihara K, Irisawa H. (1980). Inward current activated during hyperpolarization in the rabbit sinoatrial node cell. *Pflugers Arch* 385(1):11–19. doi:10.1007/bf00583909.1980.

Yanagihara K, Noma A, Irisawa H. (1980a). Reconstruction of sino-atrial node pacemaker potential based on the voltage clamp experiments. *Jpn J Physiol.* 30(6):841–857. doi:10.2170/jjphysiol.30.841.1980.
